# Comparative Efficacy of Neoadjuvant Nivolumab Plus Chemotherapy versus Conventional Comparator Treatments in Resectable Non-Small-Cell Lung Cancer: A Systematic Literature Review and Network Meta-Analysis

**DOI:** 10.3390/cancers16132492

**Published:** 2024-07-08

**Authors:** Nicolas Girard, Mariam Besada, Basia Rogula, Stefano Lucherini, Lien Vo, Mohammad A. Chaudhary, Sarah Goring, Greta Lozano-Ortega, Mia Tran, Nebibe Varol, Nathalie Waser, Winifred W. Yu, Jay M. Lee, Jonathan Spicer

**Affiliations:** 1 Department of Medical Oncology, Institut Curie, 75005 Paris, France; 2 Paris Saclay University, University of Versailles Saint-Quentin-en-Yvelines (UVSQ), 78000 Versailles, France; 3 Broadstreet HEOR, Vancouver, BC V6A 1A4, Canada; 4 Bristol Myers Squibb, Uxbridge UB8 1DH, UK; 5 Bristol Myers Squibb, Lawrenceville, NJ 08648, USA; 6 ICON Plc, Burlington, ON L7N 3G2, Canada; 7 UCLA Health, Los Angeles, CA 90095, USA; 8 Department of Thoracic Surgery, McGill University Health Center, Montreal, QC H3G 1A4, Canada

**Keywords:** resectable non-small-cell lung cancer, systematic literature review, network meta-analysis

## Abstract

**Simple Summary:**

Lung cancer is the leading cause of cancer deaths worldwide. Surgery, with or without neoadjuvant or adjuvant chemotherapy has historically been the mainstay of treatment for resectable non-small-cell lung cancer (rNSCLC). However, as many as half of those treated still die of the disease. Immune checkpoint inhibitors are changing the landscape of treatment both in the neoadjuvant and adjuvant rNSCLC settings in many countries. In this review and analysis, outcomes among patients treated in the neoadjuvant setting (i.e., prior to surgery) with the immune checkpoint inhibitor nivolumab combined with chemotherapy were compared to those of patients treated with the historical standard of care treatments, using data from published studies. The results of this evidence synthesis show that neoadjuvant nivolumab when combined with chemotherapy improves the likelihood of event-free survival and a pathological complete response for patients relative to traditional treatments, specifically, surgery, alone or in combination with neoadjuvant or adjuvant (i.e., after surgery) chemotherapy, and neoadjuvant chemoradiotherapy.

**Abstract:**

Background: This study aimed to estimate the relative efficacy of neoadjuvant nivolumab in combination with chemotherapy (neoNIVO + CT) compared to relevant treatments amongst resectable non-metastatic non-small-cell lung cancer (rNSCLC) patients. Methods: Treatment comparisons were based on a network meta-analysis (NMA) using randomized clinical trial data identified via systematic literature review (SLR). The outcomes of interest were event-free survival (EFS) and pathological complete response (pCR). NeoNIVO + CT was compared to neoadjuvant chemotherapy (neoCT), neoadjuvant chemoradiotherapy (neoCRT), adjuvant chemotherapy (adjCT), and surgery alone (S). Due to the potential for effect modification by stage, all-stage and stage-specific networks were considered. Fixed-effect (FE) and random-effects Bayesian NMA models were run (EFS = hazard ratios [HR]; pCR = odds ratios [OR]; 95% credible intervals [CrI]). Results: Sixty-one RCTs were identified (base case = 9 RCTs [*n* = 1978 patients]). In the all-stages FE model, neoNIVO + CT had statistically significant EFS improvements relative to neoCT (HR = 0.68 [95% CrI: 0.49, 0.94]), S (0.59 [0.42, 0.82]), adjCT (0.66 [0.45, 0.96]), but not relative to neoCRT (HR = 0.77 [0.52, 1.16]). NeoNIVO + CT (5 RCTs) had statistically significant higher odds of pCR relative to neoCT (OR = 12.53 [5.60, 33.82]) and neoCRT (7.15 [2.31, 24.34]). Stage-specific model findings were consistent. CONCLUSIONS: This NMA signals improved EFS and/or pCR of neoNIVO + CT relative to comparators among patients with rNSCLC.

## 1. Introduction

Lung cancer is the leading cause of cancer deaths worldwide [1]. Surgery with curative intent is the cornerstone of treatment for resectable (stage I, II and select III) non-small-cell lung cancer (rNSCLC) [2,3,4]. However, 30 to 55% of patients who undergo curative surgery have recurrence and ultimately die of their disease [5,6]. Historically, surgery can be followed by adjuvant therapy with platinum-based chemotherapy, which has been shown to improve overall survival (OS); however, the absolute 5-year survival benefit of adjuvant therapy is moderate compared with observation alone [7,8,9]. Neoadjuvant chemotherapy has also been associated with a moderate survival benefit; a 2014 meta-analysis found that rates of 5-year recurrence-free survival (RFS) with neoadjuvant chemotherapy followed by surgery were slightly higher than for surgery alone (36% vs. 30%) [10]. Radiation as an add-on to neoadjuvant chemotherapy is a standard approach in many institutions when a lobectomy is feasible; however, its clinical benefit has not been established [11,12].

The emergence of immune checkpoint inhibitors has revolutionized the treatment of various cancers, including rNSCLC [13,14,15,16,17,18,19,20,21]. Neoadjuvant treatment with nivolumab in combination with chemotherapy (neoNIVO + CT) has been approved in many countries based on findings from the randomized, open-label, phase 3 CheckMate 816 (CM816) clinical trial which compared neoNIVO + CT to neoadjuvant chemotherapy (neoCT). NeoNIVO + CT has now become the standard of care for patients with rNSCLC in many countries. Other immunotherapies (IOs) have been explored in the perioperative and adjuvant settings, including but not limited to pembrolizumab (PEMBRO), durvalumab (DURVA), and atezolizumab (ATEZO).

Given the rapidly changing treatment landscape in the rNSCLC space, which is shifting towards IO-based treatment strategies for non-oncogene addicted rNSCLC, it is important to understand the place of neoadjuvant IO relative to conventional treatments such as surgery alone (S), neoCT, adjuvant chemotherapy (adjCT), and neoadjuvant chemoradiotherapy (neoCRT). The objective of this study was to compare event-free survival (EFS), and pathological complete response (pCR) associated with neoNIVO + CT against conventional comparators amongst patients with stage IB-IIIA rNSCLC by means of a systematic literature review (SLR) and network meta-analysis (NMA).

## 2. Materials and Methods

The study was conducted according to a pre-specified research protocol developed in alignment with good practice guidelines for SLR and NMA [22,23].

### 2.1. SLR and NMA Eligibility

A systematic search of the literature was conducted using Embase, MEDLINE, and the Cochrane Central Register of Controlled trials through Ovid, complemented by a search of major conference proceedings in the last two years (Tables S1–S4). The SLR was designed to capture randomized controlled trials (RCTs) based on pre-defined broad inclusion criteria (Table S5). For the base case NMA, eligibility was limited to studies that evaluated the therapies of interest (neoNIVO + CT, neoCT, neoCRT, adjCT, S) among patient populations that were deemed resectable at study randomization (which was required to occur prior to surgery or neoadjuvant treatment); chemotherapy-based regimens were restricted to those involving 3rd generation platinum-based doublets. While perioperative IO-based regimens and adjuvant IO-based regimens were of interest, they were either not available at the time of the SLR conduct (e.g., perioperative IO), or were not relevant for the NMA due to known differences in study design (e.g., ATEZO administered after surgery and a course of adjCT; explored in a separate analysis) [24]. A detailed description of the NMA selection criteria is defined in Table S6.

Abstract and full text screening were conducted by two experienced reviewers. Arbitration was provided by a third reviewer to resolve any disagreements. Reasons for exclusion were recorded at abstract and full text screening. The study selection process was documented following the Preferred Reporting of Items for Systematic Reviews and Meta-Analyses (PRISMA) flow diagram [22]. Data extraction was conducted by a single reviewer using a customized Microsoft Excel^®^ form, and all the extracted data were independently verified and validated by a second reviewer. Kaplan–Meier (KM) curves were digitized using digitizing software (DigitizeIt v2.5.3) [25]. Included studies were critically appraised by a single reviewer using the Cochrane risk-of-bias tool for randomized trials (RoB 2) [26]. The systematic review did not register in a public registry.

### 2.2. Target Populations

Several target populations were defined for the NMA. First, a broadly defined target population was considered: patients with stage IB (tumour size ≥ 4 cm) to IIIA (AJCC TNM 7th edition) rNSCLC (hereafter referenced as IB-IIIA), which aligns with the inclusion criteria in CM816. All NMA-eligible RCTs were included in this network, under the core assumption that, within stages IB to IIIA, disease stage does not modify relative treatment effect between the regimens of interest (additional details in Table S7).

Second, more narrowly defined stage-specific target populations were considered, including: (1) an early stage (IB-II) population; (2) a stage IIIA population (spanning all relevant nodal status within stage IIIA); and (3) a stage IIIA population with mediastinal lymph node metastasis (IIIA N2 disease). NMA-eligible RCTs were included in these stage-specific networks if the eligibility criteria of the RCT—or available subgroup data—aligned with those target populations. For the stage IIIA N2 network, an exception was made for CM816, as nodal status was not recorded in this RCT; as a result, stage IIIA data (not restricted to N2) was used. The stages IB–IIIA and stage-specific models were considered base case models onto which the sensitivity analyses were applied.

The European Medicines Agency (EMA) approved neoNIVO + CT for its use among rNSCLC patients with high risk of recurrence (i.e., stage II to IIIA) and PD-L1 expression ≥1% [27]. As such, two additional PD-L1 expression level ≥1% specific populations were defined: (1) PD-L1 ≥ 1% in a stage IB-IIIA population, so as to not break randomization in CM816 (hereinafter described as PD-L1 specific), and (2) PD-L1 ≥ 1% in a stage II-IIIA population to align with the EMA indication (hereinafter described as EMA-target). Both target populations also addressed the potential for effect modification by levels of PD-L1 expression for comparisons involving IOs, as PD-L1-specific inputs were used for studies evaluating an IO-based regimen [28]. In general, inputs were aligned with the target population (i.e., subgroup-specific data were used where available).

### 2.3. Data Preparation

HRs were used to inform the EFS NMA; whenever HRs were not reported but KMs were available, HRs were estimated with Cox proportional hazard models applied to individual patient-level data (IPD) reconstructed using the methods described by Rogula et al. and Guyot et al. [29,30]. For the binary endpoint pCR, the counts of patients who achieved pCR and the total sample size in each arm were used as model inputs. For multi-arm trials involving two similar regimens (e.g., two neoCRT arms and one neoCT arm), the two arms were pooled where possible. Reconstructed IPD were used to pool EFS data, and counts were summed for pCR.

### 2.4. Quantitative Evidence Synthesis

For EFS, the assumption of proportional hazards was tested using the reconstructed IPD (Table S8). Fixed-effect (FE) and random-effects (RE) Bayesian NMA models were run (Table S8). HRs were reported for EFS, and odds ratios (OR) were reported for pCR; Bayesian 95% credible intervals (CrI) were computed. NeoCT was used as the reference treatment in the analyses. Absolute estimates of EFS at 2, 3, and 5 years were generated by applying the computed HRs to assumed hazards for the reference treatment (modeled as a lognormal parametric curve fitted to the neoCT arm from CM816). Similarly, absolute percentages of patients achieving pCR were estimated for each treatment by applying the computed ORs to the observed odds of pCR in the neoCT arm from CM816, converted from odds to percentages using standard functions.

All analyses were performed using R version 4.3.0 and WinBUGS version 1.4.3.

### 2.5. Sensitivity Analysis

Two sensitivity analyses were conducted. First, eligibility was expanded to include RCTs that randomized patients after surgical resection; this enabled the inclusion of additional evidence (e.g., both potentially resectable and completely resected patients). Second, eligible comparators were expanded to include RCTs involving 2nd generation platinum-based chemotherapies. Many of these treatments are no longer included in the National Comprehensive Cancer Network (NCCN) or European Society for Medical Oncology (ESMO) guidelines but may be used in clinical practice albeit to a lesser degree [2,3,31]. Sensitivity analyses were applied to the base case EFS models.

The full list of evaluated models is presented in Table S9.

## 3. Results

### 3.1. Evidence Base

The SLR was executed on 15 November 2022, and yielded 12,863 abstracts, of which 61 RCTs met the broader SLR eligibility criteria. From these, a total of 18 RCTs met the NMA PICOS criteria. The PRISMA diagram is presented in Figure S1. Among these, nine RCTs were included across the various target-population-specific and endpoint-specific base case models. Of note, although perioperative IO regimens were not included in the NMA PICOS for this study, the NADIM II RCT was included, as the pCR data generated following receipt of neoNIVO + CT in the study was considered relevant to the pCR models in this NMA, as the estimates are not affected by the receipt of adjuvant nivolumab. Five RCTs were added to the EFS base case set for the sensitivity analyses expanding to second generation chemotherapies (total = 13 RCTs); and four were added to the EFS base case set in the sensitivity analysis expanding to RCTs involving randomization after surgery (total = 12 RCTs). The stage-specific target populations for the base case, across various endpoints, were informed by four RCTs (stage IB-II); three RCTs (stage IIIA); and five RCTs (stage IIIA N2).

**Figure 1:**
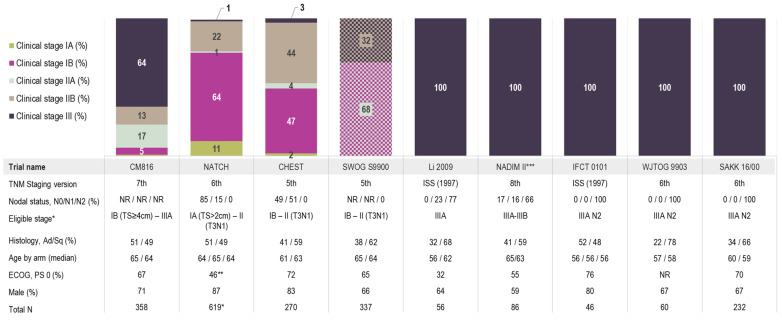
Stage distribution and key patient characteristics across the base case studies. * One patient was included in NATCH despite having stage IV disease (T4N0); ** 1% in NATCH had ECOG Performance status of 2; *** NADIM II included stage IIIA to IIIB patients based on the 8th edition for TNM staging which aligns with stage IIIA of the 7th edition as they did not include T4N2 or anyone with N3 disease. Abbreviations: PS, performance status; TS, tumour size. Note: SWOG S9900 was the only study that reported staging in two distinct groups (IB/IIA and IIB/IIIA) and as such, is visualized distinctly from the rest of the studies; 68% of patients had stage IB/IIA disease (presented in a purple and light sage cross-hatch pattern) and 32% of patients had stage IIB/III disease (presented in a light brown and dark navy cross-hatch pattern).

Characteristics of the 18 studies included in the NMA are presented in Table 1 and the baseline characteristics of the base case studies are presented in Figure 1. Median age ranged from 56 to 65 years. There was some heterogeneity in terms of the proportion of males (range: 61% to 89%), regimen characteristics (cisplatin-based or carboplatin-based regimens), number of treatment cycles (two vs. three), concurrent vs. sequential radiotherapy, squamous histology (range: 16% to 60%), stage (including staging criteria used) and post-surgical therapy amongst studies with a surgery-alone arm.

The RCTs were considered to have some concerns of bias. Seven base case RCTs were open-label, while the remaining two studies did not report their blinding status. The methods of randomization were poorly reported in some of the studies and pre-specified analysis plans were often not available, especially amongst older trials (Figure S2).

### 3.2. Proportional Hazard Assessment and Model Selection

There was no strong evidence of violation of the proportional hazard assumption of the evidence base for EFS (Figure S3). As such, an analysis relying on the assumption of a constant hazard ratio was deemed appropriate.

Due to the sparse evidence base, the between-study standard deviation could not be estimated with precision in the RE model. As such, the fixed-effect model results are presented for all endpoints and networks. The width of the 95% CrIs of the outputs from the RE model were highly influenced by the choice of prior on the between-study standard deviation; models using informed prior distributions are presented in the Tables S10–S15; Figures S4–S7.

### 3.3. Relative Efficacy for Event-Free Survival

**Figure 2:**
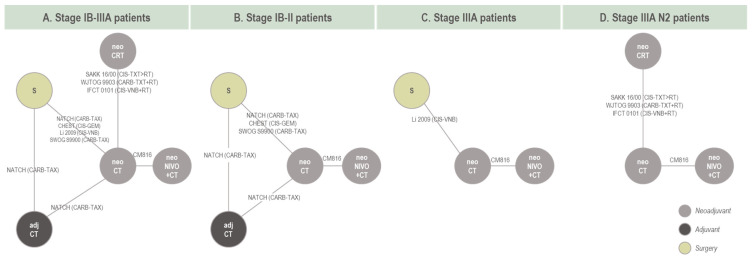
Network of evidence for base case EFS models. Note: For studies involving neoCT, neoCRT, or S, see baseline characteristics regarding protocol-defined use of post-surgical therapy. Amongst the four base case studies which included a surgery alone arm, surgery was not followed by discretionary adjCT apart from Li 2009. Both Li 2009 and NATCH were also the only two studies that allowed for adjuvant radiotherapy following surgery in some cases. EMA-target network is not reflected in this diagram; exclusions relating to the EMA-target network are available in Supplementary Material. Networks of evidence used in the sensitivity analyses are available in the Supplementary Material. Abbreviations: adj, adjuvant; CARB, carboplatin; CIS, cisplatin; CT, chemotherapy; CRT, chemoradiotherapy; GEM, gemcitabine; neo, neoadjuvant; NIVO, nivolumab; RT, radiotherapy; S, surgery; VNB, vinorelbine; TAX, paclitaxel; TXT, docetaxel.

The eight RCTs included in the analysis of EFS formed a connected network of evidence in the stage IB-IIIA target population (Figure 2A), including data from 1978 patients and 18 trial arms (17 effective arms due to the pooling of two neoCRT arms in IFCT 0101). All networks of evidence were primarily star-shaped, with one closed loop formed by one three-arm trial (NATCH) in the stage IB-IIIA and stage IB-II networks (Figure 2A,B). This network geometry precluded inconsistency assessment. Data inputs and endpoint definitions informing the EFS NMA are provided in Table S10.

**Figure 3:**
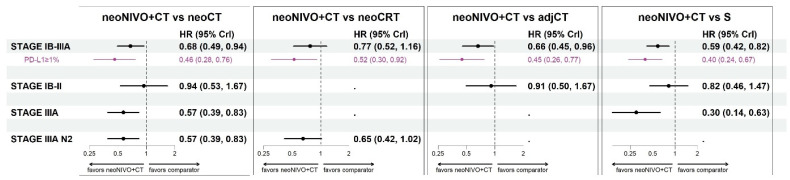
Event-free survival hazard ratio estimates for neoNIVO + CT vs. all relevant comparators across base case and the PD-L1 specific models (fixed-effect model estimates). Note: the results associated with PD-L1 ≥ 1% specific target population conducted within the stage IB-IIIA network are illustrated in purple. Sensitivity results are available in Figure S4; the EMA-target results are available in Tables S16–S18; Figures S8–S10. Abbreviations: adj, adjuvant; CrI, credible interval, CT, chemotherapy; CRT, chemoradiotherapy; HR, hazard ratio; neo, neoadjuvant; NIVO, nivolumab; S, surgery.

For the target population of patients with stage IB-IIIA, neoNIVO + CT was associated with statistically significant improvements in EFS relative to neoCT (HR = 0.68 [95% CrI: 0.49, 0.94]), S (0.59 [0.42, 0.82]), and adjCT (0.66 [0.45, 0.96]) (Figure 3). For the comparison to neoCRT, neoNIVO + CT trended towards being more efficacious but did not reach statistical significance (HR = 0.77 [0.52, 1.16]). For target populations having a more advanced stage (stage IIIA, and stage IIIA N2), the conclusions were similar for comparisons against neoCT, S and neoCRT, with stronger magnitudes of effect favouring neoNIVO + CT. The adjCT comparator was not included in these networks due to lack of available data. In the early-stage target population (IB-II), neoNIVO + CT still trended towards being more efficacious consistent with the all-stages (IB-IIIA) network (for common comparators), yet HRs were closer to the null value of 1 and did not reach statistical significance. The findings from the stage IB-IIIA PD-L1 ≥ 1% target population were relatively more favourable for neoNIVO + CT compared to the stage IB-IIIA base case model. Tabular summaries of HRs between all treatments in the stage IB-IIIA base case network are available in Table S11.

Anchored on an assumed EFS curve for the neoCT arm, absolute EFS estimates at 2-year, 3-year, and 5-year timepoints are available for each comparator in Table S12. For neoNIVO + CT, 5-year EFS ranged from 43% (stage IIIA and stage IIIA N2 target population) to 46% (stage IB-IIIA target population), whereas for S alone, 5-year EFS ranged from 6% (stage IIIA) to 38% (stage IB-II).

The findings from the sensitivity analyses were consistent with the base case models (Figure S4). The inputs and results for the EMA-target model are presented in Tables S16–S18; Figures S8–S10, and the model outputs were closely aligned with those from the PD-L1 ≥ 1% stage IB-IIIA model.

### 3.4. Relative Efficacy for Pathological Complete Response

**Figure 4:**
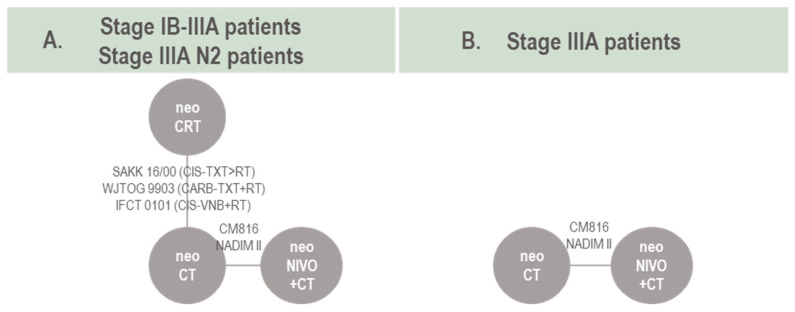
Network of evidence for pCR models. Note: for studies involving neoCT or neoCRT (e.g., neoCT vs. S) see baseline characteristics summary. NADIM II was included in the pCR analysis as the intervention arm (perioperative nivolumab combined with neoadjuvant chemotherapy) can effectively be considered comparable to neoadjuvant nivolumab combined with neoadjuvant chemotherapy as pCR outcomes are collected prior to receipt of any adjuvant therapy. Abbreviations: CARB, carboplatin; CIS, cisplatin; CT, chemotherapy; CRT, chemoradiotherapy; neo, neoadjuvant; NIVO, nivolumab; TXT, docetaxel; VNB, vinorelbine.

The networks of evidence for pCR in the stage IB-IIIA and stage IIIA N2 target population are presented in Figure 4A. As all studies comparing neoCRT vs. neoCT were conducted among patients with stage IIIA N2, the main difference between these two models was the input from CM816 (intent-to-treat vs. stage IIIA OR, respectively) and NADIM II (intent-to-treat vs. stage III N2 OR). Five RCTs informed the stage IB-IIIA/IIIA N2 network, including data from 780 patients and 11 trial arms, resulting in ten effective arms as two neoCRT arms were pooled into a single arm (IFCT 0101). For the stage IIIA target population, only two studies informed a connection between neoNIVO + CT and neoCT (Figure 4B), and therefore a pairwise meta-analysis of direct evidence was conducted. For the stage IB-II target population, no quantitative evidence synthesis was conduced as the evidence base was only informed by CM816. Data informing the pCR NMA, along with pCR definitions are available in Table S13.

**Figure 5:**
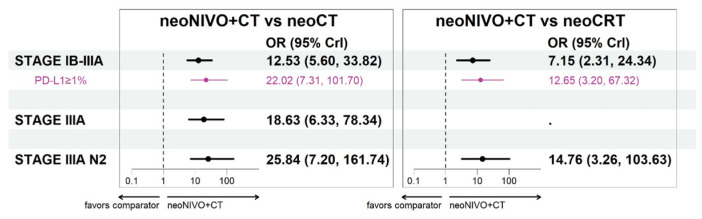
Pathological complete response odds ratio estimates for neoNIVO + CT vs. all relevant comparators across base case and the PD-L1 specific models (fixed-effect model estimates). Note: The stage IB-II cases are not represented in the figure as they were only informed by CheckMate 816 (odds ratio: 6.85; 95% confidence interval: 1.89, 24.76) and as such, no NMA was conducted for this stage. Abbreviations: CrI, credible interval; CT, chemotherapy; CRT, chemoradiotherapy; neo, neoadjuvant; NIVO, nivolumab; OR, odds ratio.

For the target population of patients with stage IB-IIIA, neoNIVO + CT was associated with improvements in pCR relative to neoCT (OR = 12.53 [5.60, 33.82]) and neoCRT (7.15 [2.31, 24.34]) (Figure 5). Estimates of relative improvement were greater in the stage IIIA and IIIA N2 target populations. Anchored on the neoCT arm of CM816, the proportions achieving pCR for neoNIVO + CT were 20%, 14% and 19% in the stage IB-IIIA, stage IIIA and stage IIIA N2 target populations, respectively (Table S14). Tabular summaries of ORs between all treatments in the stage IB-IIIA base case network are available in Table S15.

Findings from the target populations that used inputs from the PD-L1 ≥ 1% CM816 population had a larger magnitude of effect for neoNIVO + CT compared to the base case model. As shown in Tables S16–S18; Figures S8–S10, the EMA-target model closely aligned with the results of the stage IB-IIIA PD-L1 ≥ 1% model.

### 3.5. Safety

Safety outcomes were not included in the NMA due to limited data and large numbers of zero cells; for completeness, however, a tabular summary of the overall occurrence of adverse events and adverse events leading to discontinuation across the base case studies in the NMA are included in Tables S19 and S20.

## 4. Discussion

The positive findings from CM816 have established neoNIVO + CT as a new standard of care for patients with rNSCLC. Our study provides evidence of improved EFS and pCR with neoNIVO + CT relative to conventional therapies, namely neoCT, adjCT, and S alone. These results help to establish the value of a neoadjuvant IO treatment strategy and provide a benchmark against which to contrast emerging evidence from other IO-based regimens.

Results from the more narrowly defined populations suggest that the greatest clinical benefit associated with neoNIVO + CT relative to conventional therapies may be among patients with stage IIIA and patients whose tumours express PD-L1 (even though numerical benefits were also observed among patients with stage IB-II and PD-L1 < 1%) [32]. However, limitations in subgroup sizes and statistical power in CM816 warrant caution in interpreting these findings. Additional evidence from observational studies as well as a longer follow up for CM816, particularly relevant for earlier stage patients, could help elucidate which subgroups are likely to have a meaningful clinical benefit from neoNIVO + CT.

This NMA also provided additional evidence with respect to the superiority of neoadjuvant therapy relative to S alone or adjCT. It was already well understood that, while only moderately more efficacious [34], neoadjuvant chemotherapy helped overcome the limitations of adjCT, namely, higher completion rates of planned chemotherapy cycles [34,51], and avoidance of systemic treatment delays experienced by patients receiving adjCT [52,53,54]. However, in clinical practice, some physicians or patients may historically have considered bypassing neoadjuvant therapy to avoid the risk of surgery being delayed or cancelled, opting for a surgery-first strategy. The emergence of early IO therapy in the rNSCLC space has provided an opportunity to treat micrometastatic disease and enhance the immune response when bulk tumour and tumour antigens are present, and has resulted in a boost in the efficacy of the neoadjuvant treatment strategy, relative to neoCT, as demonstrated by CM816 [9,32,55]. The compelling results of this NMA have further helped establish neoNIVO + CT as a superior strategy to S alone and adjCT, in alignment with NCCN recommendations that all patients who are IO-eligible should be considered for neoadjuvant IO-based treatments.

This NMA has provided inconclusive evidence on the relative efficacy of neoNIVO + CT relative to neoCRT among patients with stage IIIA, for which neoCRT is recommended [2]. The findings within the stage-specific IIIA N2 population from the current NMA indicate that the odds of pCR are 14 times higher, and that the EFS risk is 35% lower with neoNIVO + CT relative to neoCRT; however, the corresponding estimates of EFS benefit were not statistically significant. In other indications, evidence suggests that adding radiotherapy to neoCT may reduce the risk of locoregional recurrence relative to neoCT alone, via its localized delivery, but does not reduce the risk of distant metastases [56]. In addition, no studies to date have demonstrated an OS benefit of neoCRT relative to neoCT. In terms of tolerability, the addition of radiotherapy to neoCT could potentially impact the fitness of patients to proceed to surgery after neoadjuvant treatment. For these reasons, the role of neoCRT as a treatment option for rNSCLC patients is uncertain (except among patients for which IO-based therapies are contraindicated) especially as the treatment landscape changes with the introduction of IO-based therapies.

While the relative safety of neoNIVO + CT could not be investigated via the NMA due to several challenges (AEs being reported for some agents and not others due to differences in the mechanism of action, sparse data with large numbers of zero cells, and non-reporting), evidence from CM816 indicates that the addition of nivolumab to neoCT is not associated with a higher incidence or greater severity of AEs [20]. Two of the studies in the NMA evidence base reported the proportion of patients experiencing any AEs of grades 3 to 4; SAK 16/00 reported fewer grade 3–4 CT-related AEs in the neoCRT arm (45%) than in the neoCT arm (60%) and CHEST reported fewer grade 3–4 AEs in the surgery-alone arm (11%) compared to the neoCT arm (41%) [35,40]. In addition, surgery-related AEs such as surgical 90-day morbidity/mortality and delay or cancellation of surgery due to toxicity were not captured in the current review as they were not pre-defined fields but are important to consider when interpreting the efficacy-focused findings of our analysis. In CM816, while the percentage of patients with delayed surgery was similar in the two treatment groups (21% and 18% in the neoNIVO + CT and neoCT arms, respectively), patients treated with neoNIVO + CT had shorter operations, had higher rates of minimally invasive surgery (30% vs. 22%), lower rates of pneumonectomies (17% vs. 25%) and experienced fewer conversions to open surgery (11% vs. 16%) compared to patients treated with neoCT; these differences were most notable in the stage IIIA cohort [20].

The findings presented in this analysis are consistent with other meta-analyses. For the comparison of adjCT to S, several meta-analyses have been conducted [7,51,57,58]; however, these were primarily based on evidence from RCTs that only enrolled completely resected patients, based on post-surgical eligibility assessments. Our sensitivity analysis also captured such studies, although these do not necessarily reflect the relative effect in a potentially resectable population. Disease-free or recurrence-free survival effect estimates for adjCT vs. S in those previous meta-analyses ranged from 0.67 (0.56, 0.81) to 0.84 (0.78, 0.91) depending on study eligibility, which aligns with our EFS HR sensitivity analysis estimate of 0.76 for adjCT vs. S and was stronger than the HR in our base case that excluded RCTs restricted to completely resected patients (HR of 0.89). The 2014 meta-analysis reported by the NSCLC Meta-analysis Collaborative Group estimated an RFS HR of 0.85 (95% CI: 0.76, 0.94) for the comparison of neoCT vs. S; highly consistent with the ones produced by our NMAs (0.86, 95% CrI: 0.78, 0.96) [10].

This study was not without limitations. First, there was some heterogeneity in the evidence base in terms of stage, regimen characteristics, gender distribution, and histology. With respect to stage, no conclusive evidence has been identified as to whether it conveys treatment effect modification in the rNSCLC space. However, an attempt was made to address heterogeneity through consideration of stage-specific models, but the significant evolution in clinical staging and progress in surgical techniques during the study timeline could have led to discrepancies in the baseline stage inclusions that do not completely match current understanding of rNSCLC. It was not possible to control for these important variables. With respect to regimen characteristics, gender distribution and histology, while these characteristics may be prognostic among patients with rNSCLC, they are not known treatment-effect modifiers, so are unlikely to have introduced bias to the NMA. Future work can be conducted using a multi-level network meta-regression, as described by Phillippo et al. 2020, to adjust for all heterogeneous prognostic and effect-modifying variables in the evidence base [59]. Second, the base case NMA was informed by a small number of studies, with the largest network of evidence being informed by at most three studies per available connection. Some of the analyses presented relied on subgroup data (e.g., stage-specific data from all-stage trials). It is important to note that these studies may not be powered to detect treatment effects amongst these subgroups. In addition, if trials were not stratified by these subgroups upon randomization, patient characteristics may not necessarily be balanced between study arms within these subgroups, which may introduce bias. For both reasons, analyses relating to the specific target populations should be interpreted with caution. Third, due to the paucity of data amongst the base case trials, relevant outcomes such as major pathologic response (MPR), could not be included in this evidence synthesis. In the case of pCR, where there were limited data, an indirect comparison versus neoCRT could not be drawn for the stage IB-II, or stage IIIA target populations; however, neoCRT is most relevant to the target population of stage IIIA N2 for which an indirect comparison was possible. Similarly, and perhaps most notably, OS was not evaluated as it is an ongoing endpoint in CM816 and will be evaluated at a later time; however, there is evidence to suggest a positive association between pCR or EFS with OS [60,61,62,63,64,65]. Fourth, within the stage IIIA N2 specific analysis, nodal status was not captured in one study (CM816), as such, stage IIIA data were used as inputs in this model. Additionally, N2 patients are considered to be a heterogeneous population [66], and the studies informing the N2-specific models seldomly reported details related to type of N2 disease (i.e., single station vs. multi-station). Similarly, heterogeneity could have been introduced due to the complexity of defining the criteria for ‘potentially resectable’ particularly among IIIA N2 patients. In a study conducted by Mainguene et al. 2022, concordance of defining resectability was 70% between two different multidisciplinary tumour board sessions, further highlighting the need for more specific assessment criteria [66]. This known lack of concordance could lead to differences in the proportion of patients across trials that are less likely to respond to treatment (i.e., those who could have met non-resectability criteria); these differences can result in biased relative effect estimates that could not be measured or adjusted for. Finally, while other immunotherapy regimens such as ATEZO, DURVA, and PEMBRO are rapidly changing the treatment landscape, they were not included in this analysis because they were not approved and/or were not relevant due to known differences in study conduct [67,68,69,70]. Future analyses to include comparisons with adjuvant IO-therapies will need to adjust for differences in their study designs and differences in enrolled patient populations. Methodology has been proposed to reduce biases associated with differences in trial design with respect to the time of randomization and patient eligibility, such as those between CM816 and IMpower010/Keynote-091 [24]. Similarly, perioperative immunotherapy, including perioperative NIVO was not included as a comparator of interest also due to lack of regulatory approval at the time of analysis. The findings from CM816 and our NMA provide supporting evidence that early introduction of IO can mount an immune system response while the tumour is still present. Findings from CM816 have motivated the exploration of perioperative IO strategies, as evaluated in NADIM II (which was included in our base case pCR analysis) and CheckMate 77T (an ongoing clinical trial of perioperative NIVO plus neoCT published after the SLR cut-off date). NADIM II found that perioperative NIVO combined with neoCT reduced the risk of PFS by 53% compared to neoCT alone [71]. CM77T demonstrated statistically significant EFS improvement with perioperative NIVO combined with neoCT versus neoCT alone (HR: 0.58; 95% CI: 0.42–0.81) [72]. It will be important for future analyses to compare perioperative IO regimens with established treatments in rNSCLC, including neoNIVO + CT to help elucidate the clinical relevance of the additional adjuvant immunotherapy component.

## 5. Conclusions

This NMA provides evidence that neoNIVO + CT confers added clinical benefit in terms of EFS and pCR relative to neoCT, S, and adjCT amongst patients with rNSCLC. These findings are restricted by limited data, including the shorter follow-up from CM816. Updates to this analysis will be conducted with more mature data from CM816, which may mitigate some of the uncertainty associated with the estimates. Similarly, the impact of neoNIVO + CT on OS in relation to existing treatment options will need to be assessed once mature data are available.

## Figures and Tables

**Table 1:** Characteristics of included studies.

Study (Author Year) *	N	Arms (n)	Phase	Blinding	Median FU (yrs)	Region	Post-Surgical Treatment Permitted	
							adjCT	adjRT
Base case studies								
CM816 (Forde 2022) [20,32]	358	neoCT (179), neoNIVO + CT (179)	3	Open-label	3.5	International	Yes (investigator discretion)	Yes (investigator discretion)
NADIM II (Provencio 2022) [33]	86	neoCT (29), periNIVO + neoCT ^◆^ (57)	2	Open-label	-	Spain	No	No
NATCH (Felip 2010) [34]	619	S (210), neoCT (199), adjCT (210)	3	Open-label	4.2	Europe	No	Yes (if pN2)
CHEST (Scagliotti 2012) [35]	270	neoCT (129), S (141)	3	-	2.6–3.3 ^‡^	Europe	No **	No **
SWOG S9900 (Pisters 2010) [36]	337	neoCT (169), S (168)	3	Open-label	5.3	US and Canada	No **	No **
Li 2009 [37]	56 ^†^	neoCT (28), S (28)	3	Open-label	3.2	China	Yes, all patients	Yes, R1 or R2
IFCT 0101 (Girard 2010) [38]	46 ^†^	neoCT (14), neoCRT ^◆◆^ (17), neoCRT ^◆◆^ (15)	2	Open-label	2.6	France	Yes, R2 ***	Yes, R1 or R2 ***
WJTOG 9903 (Katakami 2012) [39]	60 ^†^	neoCT (29), neoCRT ^◆◆^ (31)	3	-	5.1	Japan	No	Unclear
SAKK 16/00 (Pless 2015) [40]	232	neoCT (115), neoCRT ^◆◆^ (117)	3	Open-label	4.4	Europe	Unclear	Yes, R1 or R2
Studies added to the sensitivity analyses (3rd and 2nd generation chemotherapies)								
Chen 2013 [41]	337	neoCT (169), S (168)	-	Open-label	4.5	China	Yes, all patients	Yes, if pN2
IFCT 0001 (Depierre 2002) [42]	355	neoCT (179), S (176)	3	-	6.7	France	Yes, responders in S arm	Yes, R1 or R2 of pT3 or pN2
MRC LU22 (Gilligan 2007) [43]	519	neoCT (258), S (261)	3	-	-	Europe	Possible, if deemed inoperable at time of surgery or on progression	
JCOG 9209 (Nagai 2003) [44]	62 ^†^	neoCT (31), S (31)	3	Open-label	6.2	Japan	No	Yes, R1 or R2
Rosell 1994 [45,46]	60	neoCT (30), S (30)	-	Open-label	5.0	Spain	Unclear	Yes, all patients
Studies added to the sensitivity analyses (completely resected patients)								
JBR10 (Winton 2005) [8,47]	482	S (240), adjCT (242)	3	Open-label	9.3	Canada, US	Unclear in S arm	Unclear
ANITA (Douillard 2006) [48]	840	S (433), adjCT (407)	3	Open-label	6.3	International	Unclear in S arm	Yes (investigator discretion)
Ou 2010 [49]	150	adjCT (79), S (71)	-	-	2.4	China	Unclear in S arm	No
CALGB 9633 (Strauss 2008) [50]	344	adjCT (173), S (171)	-	Open-label	6.2	US	Unclear in S arm	Unclear

* Author and year were reported for the primary publication (the first full text published on a given trial; in addition, if no trial name was identified, author and year were used as the trial name; ^◆^ although perioperative nivolumab combined with neoadjuvant chemotherapy is not a comparator of interest, NADIM II was included in the pCR analysis as the intervention arm can effectively be considered comparable to neoadjuvant nivolumab combined with neoadjuvant chemotherapy for this endpoint. ^†^ Prematurely terminated due to slow accrual; ^‡^ stopped early due to positive results of adjuvant RCTs; ** as reported in 2014 meta-analysis by the NSCLC Meta-analysis Collaborative Group; *** at the discretion of the investigator amongst patients randomized to CRT arm; ^◆◆^ radiotherapy component was delivered sequentially in Pless 2015 and concurrently in Girard 2010 and Katakami 2012. Abbreviations: adj, adjuvant; CT, chemotherapy; CRT, chemoradiotherapy; FU, follow-up; neo, neoadjuvant; NIVO, nivolumab; peri, perioperative; S, surgery; yrs, years.

## Data Availability

The datasets produced during this study are available from the corresponding author upon reasonable request.

## Supplementary Materials

The following supporting information can be downloaded at: https://www.mdpi.com/article/10.3390/cancers16132492/s1, Table S1: Search strategy (MEDLINE)—Database(s): Ovid MEDLINE(R) and Epub Ahead of Print, In-Process and Other Non-Indexed Citations and Daily. Search was last run on 15 November 2022; Table S2: Search strategy (EMBASE)—Database(s): Embase. Search was last run on 15 November 2022; Table S3: Search strategy (Cochrane)—Database(s): EBM Reviews—Cochrane Central Register of Controlled Trials. Search was last run on 15 November 2022; Table S4: Conferences searched; Table S5: SLR PICOS; Table S6: NMA PICOS; Table S7: Evidence for and against effect modification of stage on relative effect size; Table S8: Methods for evidence synthesis; Table S9: Full list of models; Figure S1: PRISMA; Figure S2: Risk of bias assessment; Figure S3: Kaplan–Meier curves of EFS for base case: All stages, potentially resectable, third generation chemotherapies; Table S10: Data inputs and definitions for event-free survival in the base case; Table S11: Pairwise event-free survival hazard ratio estimates in the base case stage IB-IIIA model (fixed-effect model estimates); Table S12: Event-free survival percentages at 2, 3, and 5 years anchored on neoCT in the base case models (fixed-effect model estimates); Figure S4: Event-free survival hazard ratio estimates for neoNIVO + CT vs. all relevant comparators across all models (base case and sensitivity models); Figure S5: Network of evidence for EFS models with studies included in the sensitivity analyses; Figure S6: Event-free survival hazard ratio estimates for neoNIVO + CT vs. all relevant comparators across the base case and the PD-L1 specific models (random-effects model estimates); Table S13: Data inputs and definitions for pathological complete response in the base case; Table S14: Pathological complete response percentages anchored on neoCT in the base case models (fixed effect model estimates); Table S15: Pairwise pathological complete response odds ratio estimates in the base case stage IB-III model (fixed-effect model estimates); Figure S7: Pathological complete response odd ratio estimates for neoNIVO + CT vs. all relevant comparators across the base case and the PD-L1 specific models (random-effects model estimates); Table S16: Baseline patient characteristics across EFS evidence base for trials included in the stage II-IIIA PD ≥ 1% model; Table S17—EFS NMA inputs evidence base for stage II-IIIA PD ≥ 1% model; Figure S8: Event-free survival hazard ratio estimates for neoNIVO + CT vs. all relevant comparators for stage II-IIIA PD ≥ 1% model (fixed-effect model estimates); Figure S9: Event-free survival hazard ratio estimates for neoNIVO + CT vs. all relevant comparators for stage II-IIIA PD ≥ 1% model (random-effects model estimates); Table S18: Pathological complete response NMA inputs evidence base for stage II-IIIA PD ≥ 1% model; Figure S10: Pathological complete response odds ratio estimates for neoNIVO + CT vs. all relevant comparators for stage II-IIIA PD ≥ 1% model (fixed-effect model estimates); Table S19: Proportion of patients with grade 3 to 4 adverse events; Table S20: Proportion of patients with AEs leading to treatment discontinuation. References [7,8,10,20,32,33,34,35,36,37,38,39,40,41,42,43,44,45,46,47,48,49,50,52,58,73] are also cited in the supplementary materials.
