# Correction: Girard et al. Comparative Efficacy of Neoadjuvant Nivolumab Plus Chemotherapy versus Conventional Comparator Treatments in Resectable Non-Small-Cell Lung Cancer: A Systematic Literature Review and Network Meta-Analysis. *Cancers* 2024, *16*, 2492

**DOI:** 10.3390/cancers17040674

**Published:** 2025-02-17

**Authors:** Nicolas Girard, Mariam Besada, Basia Rogula, Stefano Lucherini, Lien Vo, Mohammad A. Chaudhary, Sarah Goring, Greta Lozano-Ortega, Mia Tran, Nebibe Varol, Nathalie Waser, Winifred W. Yu, Jay M. Lee, Jonathan Spicer

**Affiliations:** 1 Department of Medical Oncology, Institut Curie, 75005 Paris, France; nicolas.girard2@curie.fr; 2 Paris Saclay University, University of Versailles Saint-Quentin-en-Yvelines (UVSQ), 78000 Versailles, France; 3 Broadstreet HEOR, Vancouver, BC V6A 1A4, Canada; mbesada@broadstreetheor.com (M.B.); brogula@broadstreetheor.com (B.R.); sarah@goring.com (S.G.); glozano-ortega@broadstreetheor.com (G.L.-O.); 4 Bristol Myers Squibb, Uxbridge UB8 1DH, UK; stefano.lucherini@bms.com (S.L.); nebibe.varol@bms.com (N.V.); 5 Bristol Myers Squibb, Lawrenceville, NJ 08648, USA; lien.vo@bms.com (L.V.); ashraf.chaudhary@bms.com (M.A.C.); mia.tran@bms.com (M.T.); 6 ICON Plc, Burlington, ON L7N 3G2, Canada; nathalie.waser@iconplc.com (N.W.); winifredwyu@gmail.com (W.W.Y.); 7 UCLA Health, Los Angeles, CA 90095, USA; jaymoonlee@mednet.ucla.edu; 8 Department of Thoracic Surgery, McGill University Health Center, Montreal, QC H3G 1A4, Canada

## Addition of an Author

Winifred W. Yu was not included as an author in the original publication [1]. The corrected Author Contributions Statement is shown here. The authors apologize for any inconvenience caused and state that the scientific conclusions are unaffected. This correction was approved by the Academic Editor. The original publication has also been updated.

**Author Contributions:** N.G. provided clinical expertise and oversight for the study. M.B., S.G. and G.L.-O. contributed to the writing and to the design and interpretation of the analysis. B.R. performed the data analysis and contributed to the writing, design, and interpretation of the analysis. S.L., L.V. and M.A.C. contextualized the findings and contributed to the design and interpretation of the analysis. M.T., N.V., J.M.L. and J.S. provided clinical insights and contextualized the findings. N.W. and W.W.Y. conducted the systematic literature review. All authors have read and agreed to the published version of the manuscript.

## References

[1] Girard N., Besada M., Rogula B., Lucherini S., Vo L., Chaudhary M.A., Goring S., Lozano-Ortega G., Tran M., Varol N. (2024). Comparative Efficacy of Neoadjuvant Nivolumab Plus Chemotherapy versus Conventional Comparator Treatments in Resectable Non-Small-Cell Lung Cancer: A Systematic Literature Review and Network Meta-Analysis. Cancers.

