# Supplementary material for: Comparative Efficacy of Neoadjuvant Nivolumab Plus Chemotherapy versus Conventional Comparator Treatments in Resectable Non-Small-Cell Lung Cancer: A Systematic Literature Review and Network Meta-Analysis
Source: Cancers (Basel). 2024 Jul 8;16(13):2492. doi: 10.3390/cancers16132492 (PMC11240383; doi:10.3390/cancers16132492)
Supplement: Supplementary file 1 [file cancers-16-02492-s001.zip › cancers-3050908-supplementary.pdf]

Supplementary material A: Methods

Section A.1. Systematic review conduct

Supplementary table S1 – Search strategy (MEDLINE) - Database(s): Ovid MEDLINE(R) and Epub Ahead of Print, In-Process & Other Non-Indexed Citations and Daily. Search was last run on November 15 2022

#	Searches
1	exp Lung Neoplasms/
2	(lung* adj3 (canc* or carcinoma* or tumo?r* or neoplas*)).tw.
3	("non small cell" or "nonsmall cell").tw.
4	(1 or 2) and 3
5	Carcinoma, Non-Small-Cell Lung/
6	NSCLC.tw.
7	or/4-6
8	exp chemotherapy, adjuvant/
9	exp chemoradiotherapy, adjuvant/
10	exp radiotherapy, adjuvant/
11	exp neoadjuvant therapy/
12	(adjuvant or neoadjuvant or periadjuvant).tw.
13	or/8-12
14	exp Carcinoma, Non-small-cell Lung/su
15	exp Lung neoplasms/su
16	exp pneumonectomy/
17	(operative or preoperative or postoperative or operable or surger* or surgical* or resect* or pneumonectom*).tw.
18	exp induction chemotherapy/
19	exp combined modality therapy/
20	exp chemoradiotherapy/
21	exp radiotherapy/
22	(((chemo or radio or immuno or target* or induction) adj (therap* or treatmen*)) or chemotherap* or radiotherap* or chemoradiotherap* or radiochemotherap* or immunotherap*).tw.
23	or/18-22
24	(14 or 15 or 16 or 17) and 23
25	13 or 24
26	7 and 25
27	Epidemiologic studies/
28	exp case control studies/
29	exp cohort studies/
30	Case control.tw.
31	(cohort adj (study or studies)).tw.
32	Cohort analy$.tw.
33	(Follow up adj (study or studies)).tw.
34	(observational adj (study or studies)).tw.
35	Longitudinal.tw.

#	Searches
36	Retrospective.tw.
37	Cross sectional.tw.
38	Cross-sectional studies/
39	(single arm adj (study or studies)).tw.
40	(treatment adj2 (pattern* or path* or paradigm)).tw.
41	or/27-40
42	Randomized controlled trials as Topic/
43	Randomized controlled trial/
44	Random allocation/
45	Double blind method/
46	Single blind method/
47	Clinical trial/
48	clinical trial, phase i.pt.
49	clinical trial, phase ii.pt.
50	clinical trial, phase iii.pt.
51	clinical trial, phase iv.pt.
52	controlled clinical trial.pt.
53	randomized controlled trial.pt.
54	multicenter study.pt.
55	clinical trial.pt.
56	exp Clinical Trials as topic/
57	or/42-56
58	(clinical adj trial$).tw.
59	((singl$ or doubl$ or tripl$ or treb$) adj (blind$3 or mask$3)).tw.
60	Placebos/
61	Placebo$.tw.
62	randomly allocated.tw.
63	(allocated adj2 random$).tw.
64	or/58-63
65	57 or 64
66	Case report.tw.
67	Letter/
68	Historical article/
69	or/66-68
70	65 not 69
71	41 or 70
72	26 and 71

Supplementary table S2 – Search strategy (EMBASE) - Database(s): Embase. Search was last run on November 15 2022

#	Searches
1	exp lung tumor/
2	(lung* adj3 (canc* or carcinoma* or tumo?r* or neoplas*)).tw.

#	Searches
3	("non small cell" or "nonsmall cell").tw.
4	(1 or 2) and 3
5	exp non small cell lung cancer/
6	NSCLC.tw.
7	or/4-6
8	exp adjuvant therapy/
9	exp neoadjuvant therapy/
10	(adjuvant or neoadjuvant or periadjuvant).tw.
11	or/8-10
12	exp non small cell lung cancer/su
13	exp lung tumor/su
14	exp lung resection/
15	(operative or preoperative or postoperative or operable or surger* or surgical* or resect* or pneumonectom*).tw.
16	exp induction chemotherapy/
17	exp multimodality cancer therapy/
18	exp chemoradiotherapy/
19	exp radiotherapy/
20	(((chemo or radio or immuno or target* or induction) adj (therap* or treatmen*)) or chemotherap* or radiotherap* or chemoradiotherap* or radiochemotherap* or immunotherap*).tw.
21	or/16-20
22	(12 or 13 or 14 or 15) and 21
23	11 or 22
24	7 and 23
25	Clinical study/
26	Case control study/
27	Family study/
28	Longitudinal study/
29	Retrospective study/
30	Prospective study/
31	Randomized controlled trials/
32	30 not 31
33	Cohort analysis/
34	(Cohort adj (study or studies)).mp.
35	(Case control adj (study or studies)).tw.
36	(follow up adj (study or studies)).tw.
37	(observational adj (study or studies)).tw.
38	(epidemiologic$ adj (study or studies)).tw.
39	(cross sectional adj (study or studies)).tw.
40	(single arm adj (study or studies)).tw.
41	(treatment adj2 (pattern* or path* or paradigm)).tw.
42	or/25-29,32-41
43	Clinical trial/
44	Randomized controlled trial/

#	Searches
45	controlled clinical trial/
46	multicenter study/
47	Phase 3 clinical trial/
48	Phase 4 clinical trial/
49	exp Randomization/
50	Single blind procedure/
51	Double blind procedure/
52	Crossover procedure/
53	Placebo/
54	Randomi?ed controlled trial$.tw.
55	Rct.tw.
56	(random$ adj2 allocat$).tw.
57	Single blind$.tw.
58	Double blind$.tw.
59	((treble or triple) adj blind$).tw.
60	Placebo$.tw.
61	Prospective study/
62	or/43-61
63	Case study/
64	Case report.tw.
65	Abstract report/ or letter/
66	Conference proceeding.pt.
67	Conference abstract.pt.
68	Editorial.pt.
69	Letter.pt.
70	Note.pt.
71	or/63-70
72	62 not 71
73	42 or 72
74	24 and 73
75	conference.so.
76	conference paper/
77	conference.pt.
78	or/75-77
79	74 not 78

Supplementary table S3 – Search strategy (Cochrane) - Database(s): EBM Reviews - Cochrane Central Register of Controlled Trials. Search was last run on November 15, 2022

#	Searches
1	exp Lung Neoplasms/
2	(lung* adj3 (canc* or carcinoma* or tumo?r* or neoplas*)).tw.

#	Searches
3	("non small cell" or "nonsmall cell").tw.
4	(1 or 2) and 3
5	Carcinoma, Non-small-cell Lung/
6	NSCLC.tw.
7	or/4-6
8	exp chemotherapy, adjuvant/
9	exp chemoradiotherapy, adjuvant/
10	exp radiotherapy, adjuvant/
11	exp neoadjuvant therapy/
12	(adjuvant or neoadjuvant or periadjuvant).tw.
13	or/8-12
14	exp Carcinoma, Non-small-cell Lung/su
15	exp Lung neoplasms/su
16	exp pneumonectomy/
17	(operative or preoperative or postoperative or operable or surger* or surgical* or resect* or pneumonectom*).tw.
18	exp induction chemotherapy/
19	exp combined modality therapy/
20	exp chemoradiotherapy/
21	exp radiotherapy/
22	(((chemo or radio or immuno or target* or induction) adj (therap* or treatmen*)) or chemotherap* or radiotherap* or chemoradiotherap* or radiochemotherap* or immunotherap*).tw.
23	or/18-22
24	(14 or 15 or 16 or 17) and 23
25	13 or 24
26	7 and 25
27	conference.so.
28	conference abstract.pt.
29	27 or 28
30	26 not 29

Supplementary table S4 – Conferences searched

Meeting	2022	2021	2020
IASLC/ESMO/ELCC	✓	✓	✓
AACR	✓	✓	✓
ASCO	✓	✓	✓
SITC	×	✓	✓
ESMO Congress	×	✓	✓
IASLC WCLC	×	✓	✓
IASLC WCLC-Europe	×	✓	✓
ESMO Asia Congress	×	✓	✓

Abbreviations*: AACR: American Association for Cancer Research; ASCO: American Society of Clinical Oncology; ELCC: European Lung Cancer Congress; ESMO: European Society for Medical Oncology; IASLC: International Association for the Study of Lung Cancer; SITC: Society for Immunotherapy of Cancer; WCLC: World conference on lung cancer.*

Supplementary table S5 – SLR PICOS

	ELIGIBILITY PARAMETERS
**Population**	• Adult patients with resectable non-metastatic (stages I-IIIA) NSCLC<br>*Additional exclusions: the population entirely EGFR/ALK mutated, elderly, and performance status criterion > 2*
**Intervention/comparator**	• Surgery + chemotherapy (with or without RT)<br>• Surgery + targeted therapy (with or without RT)<br>• Surgery + immunotherapy (with or without RT)<br>• Surgery + BSC/no treatment (with or without RT)<br><br>Details of therapies allowed:<br>○ Chemotherapy:<br>– Platinum-based chemotherapy or mix of platinum-based chemotherapies[§]<br>– Oral fluorouracil therapies (i.e. S-1 and UFT in monotherapy or combination therapy)<br>– Mono-chemotherapy (e.g. docetaxel)<br>– Non-platinum-based combination chemotherapies<br>○ Targeted:<br>– Bevacizumab-based chemotherapy<br>– Gefitinib and erlotinib*<br>○ Immunotherapy<br>– Nivolumab<br>– Durvalumab<br>– Pembrolizumab<br>– Atezolizumab<br>*Exclusions: platinum-based adjuvant chemotherapy including at least one 1st generation therapy** (e.g. cyclophosphamide, doxorubicin). Experimental agents (e.g. pazopanib, panitumumab)*
**Outcomes**	• Response<br>○ Radiographic or clinical response (CR, PR, SD, and PD)<br>○ Pathologic response (pCR or MPR)<br>• Survival outcomes<br>○ OS, PFS, EFS, RFS and DFS<br>• Adverse events<br>• HRQoL
**Study design**	Interventional studies (RCTs)
**Restrictions**	English language

***Abbreviations**: BSC: Best supportive care; CR: Complete response; DFS: Disease free survival; EFS: Event free survival; HRQoL: Health-related quality of life; MPR: Major pathological response; OS: Overall survival; pCR: Pathological complete response; PD: Progressive disease; PFS: Progression free survival; PICOS: Population, intervention, comparator, outcomes, and study design; PR: Partial response; PRO: Patient reported outcome; PS: Performance status; RCT: Randomized-controlled trial; RFS: Recurrence-free survival; RT: Radiotherapy; SD: Stable disease; TKI: Tyrosine kinas inhibitor*

**targeted therapies include oncogene-targeted therapies such as TKIs and also agents like bevacizumab*

***1st generation-based chemotherapies were included if they were used in the neoadjuvant setting*

Supplementary table S6 – NMA PICOS

	ELIGIBILITY PARAMETERS
**Population**	Studies enrolling adult patients with resectable[a] nmNSCLC (stage IB-IIIA[b]).<br><br>***Sensitivity analysis only**[c]: Studies in which eligibility was assessed after surgical resection, and the study was restricted to adult nmNSCLC patients with resection*
**Intervention/comparator**	Studies comparing two or more of the following regimens:<br>1. Neoadjuvant nivolumab in combination with a 3rd generation platinum doublet[d,e,f]<br>2. NeoCT (3rd generation platinum doublet[d])[e]<br>3. NeoCRT (3rd generation platinum doublet[d]; delivered concurrently or sequentially with radiotherapy)[e]<br>4. AdjCT (3rd generation platinum doublet[d])<br>5. Surgery alone

	Sensitivity analysis only [c] *: eligible platinum CTs are expanded to include 2nd generation platinum combinationse and UFT monotherapy*<br><br>*Studies not comparing two or more of the above listed regimens will be excluded (e.g. neoCT vs another neoCT).*<br><br>*Examples of regimens not considered relevant comparators include: S-1 monotherapy, S-1 in combination with chemotherapy; UFT in combination with chemotherapy; bevacizumab-based therapy; gefitinib, 3rd or 2nd generation chemotherapies used as monotherapy; chemotherapy involving a 1st generation agent* [g]
**Outcomes**	Studies reporting at least one of the following outcomes will be retained:<br>• Survival outcomes OS, PFS, EFS, RFS, DFS, reported as relative treatment effects using a time-to-event endpoint and/or providing Kaplan-Meier plots<br>• Pathological response (pCR, MPR) to neoadjuvant therapy, reported in at least two study arms<br>• Locoregional recurrence or distant metastases reported in at least two study arms [h]
**Study design**	Randomized controlled trials

[a] *Based on study designs where patients were enrolled prior to surgery, with the intention of surgical resection*

[b] *In trials that include other stages (i.e. stage IA or stage IIIB), no more than 20% of the trial population could represent stages that were not of interest, otherwise such trials were ineligible unless they reported subgroup data. Of important note, although the CM816 population included stage IB patients with tumour sizes ≥4cm, this tumour size criterion was not applied in the PICOS as this was not consistently reported across trials.*

[c] *Studies that have features from both the population and intervention sensitivity analyses (e.g. a study conducted amongst completely resected patients evaluating 2nd generation therapy) will be excluded.*

[d] *Cisplatin or carboplatin in combination with gemcitabine (GEM), vinorelbine (VNB), paclitaxel (TAX), docetaxel (TXT), or pemetrexed (PEMX). Note, if a trial arm included a mix of treatments (e.g., 3rd and 2nd generation platinum-based chemotherapies), at least 80% of the patients in the trial should have received 3rd generation platinum-based chemotherapies for the study to be included in the base case analysis).*

[e] *In studies involving neoCT, or neoCRT (including e.g., neoCT vs S), additional administration of post-surgical CT and/or RT was permitted. Post-surgical CT and/or RT could differ across treatment arms, if administered to only a subset of patients (e.g., post-surgical CT given to responders to neoCT; or CT and/or RT given to patients with a R1 or R2 resection). However, if protocol-defined CT and/or RT was administered to all patients -- irrespective of surgical outcome or response to initial therapy-- the same post-surgical regimen must have been used in all relevant trial arms being compared, otherwise such trials were ineligible.*

[f] *Neoadjuvant nivolumab in combination with 3rd generation chemotherapy followed by post-surgical nivolumab was permitted, but only for endpoints evaluated prior to receipt of post-surgical nivolumab (i.e., pathological complete response)*

[g] *First generation chemotherapies include: methotrexate (MTX), cyclophosphamide (CYCLO), vincristine (VNC), and doxorubicin (DXR)*

[h] *Locoregional recurrences and distant metastases were not included in the PICOS of the SLR informing the NMA (although these data were extracted where available); however, given their relevance to the cost-effectiveness analysis, these outcomes were included in the NMA PICOS.*

Abbreviations*: adj, adjuvant; CT, chemotherapy; CRT, chemoradiotherapy; DFS, disease-free survival; EFS, event-free survival; MPR, major pathological response; neo, neoadjuvant; nmNSCLC, non-metastatic non-small cell lung cancer; OS, overall survival; pCR, pathological complete response; PFS, progression free survival; RFS, recurrence-free survival; RT, radiotherapy.*

Section A.2. Evidence synthesis conduct

Supplementary table S7 – Evidence for and against effect modification of stage on relative effect size

EVIDENCE FOR EFFECT MODIFICATION	EVIDENCE AGAINST EFFECT MODIFICATION
**In the adjuvant setting,**<br>2008 LACE meta-analysis of five phase 3 trials (Butts 2010; Douillard 2006; International Adjuvant Lung Cancer Trial Collaborative Group 2004; Scagliotti 2003; Waller 2004) found that there was no OS and DFS benefit in stage I patients, but significant benefit in stage II and III patients.[58]	**In the adjuvant setting,**<br>A meta-analysis of 26 trials did not find evidence of effect modification as data was limited in stage IA patients.[7]
**In the neoadjuvant setting,**<br>Within this 2023 NMA evidence base, effect sizes were stronger among patient with stage III disease (excluding N2) than among patients with stage IB-II.	**In the neoadjuvant setting,**<br>A meta-analysis of interaction terms for neoCT as compared to S was conducted for stage and found that the effect modification term was not statistically significant. [10]
**Based on expert opinion,**<br>During a clinical advisory board meeting conducted in April 2022, there was broad agreement amongst the seven participating clinicians that relative effect size is influenced by disease stage.	

Supplementary table S8 – Methods for evidence synthesis

ASSESSMENT OF PROPORTIONAL HAZARDS

To test the proportional hazards assumption, IPD were reconstructed from KM curves. The proportional hazards assumption was then evaluated through a visual inspection of the KM data, log cumulative hazard plots, Schoenfeld residuals, and Grambsch-Therneau tests.

BAYESIAN NMA APPROACH

The NMA was conducted in alignment with methodological recommendations by NICE in the UK. [52] Details on NMA model specifications are provided in the sections below.

Time-to-event data (EFS)

Observed data were included in the model using a normal likelihood as follows:

$$\log\left(h(t)_{i,k,b}\right) \sim N\left(\beta_{i,k} - \beta_{i,b}, se_{i,k,b}^2\right)$$

Where $se_{i,k}$ is the standard error of the log HR, $\log\,(h(t)_{i,k})$, from study *i* comparing treatments *k* and b.

The treatment effect model has a linear regression structure with the predicted log HR, $\log\,(h(t)_{i,k,b})$, equal to the sum of a fixed study level baseline term and the difference between the two treatment coefficients:

$$\log\,(h(t)_{i,k}) = \alpha_i + \beta_k - \beta_b$$

The fixed study level 'baseline' term is a nuisance parameter included to ensure that the treatment effect estimates are informed by within trial differences between treatment arms only. A vague prior $\alpha \sim N(0, 10^4)$ for the study specific baseline was used to guarantee the treatment effect differences are informed by the relative treatment effects between arms and not absolute treatment effects. The treatment effect coefficients (β) represent the estimated log HR of treatment *k* compared b*.* A vague prior $\beta \sim N(0, 10^4)$ was used for the treatment effects coefficients.

The corresponding random effects model substitutes the constant treatment effect with the study specific treatment effect $\delta_{i,k}$. This is normally distributed with mean $mb_{i,k} = (\beta_{i,k} - \beta_{i,b})$ and variance σ^2, where σ^2 is the random effect variance and assumed to be constant across all treatment comparisons. The modifications for the random effects model is as follows:

$$\log\,(h(t)_{i,k}) = \alpha_i + \delta_{i,k}$$

$$\delta_{i,k} \sim N\left(mb_{i,k}, \sigma^2\right)$$

$$mb_{i,k} = (\beta_{i,k} - \beta_{i,b})$$

Binary Data (pCR)

For pCR, the counts of patients who achieved pCR and the total sample size in each arm were used as model input. The underlying model was that of a logistic regression. Observed data were included using a binomial likelihood where the probability (p) of achieving pCR for study

i and treatment k is as follows:

$$r_{i,k} \sim Binomial(p_{i,k}, n_{i,k})$$

Where $r_{i,k}$ is the number of events in treatment arm k of study i, and $n_{i,k}$ is the total number of subjects in treatment arm k of study i.

Treatments k included in the model were indexed as positive integers with the baseline treatment (b) being the lowest index treatment in study i. A logit link function is used in the fixed-effect model such that for treatment arm k of study i:

$$\text{logit}(p_{i,k}) = \alpha_i + (\beta_k - \beta_{i,b})$$

Where α_i is the study-specific baseline term, and $\beta_k - \beta_{i,b}$ is the study-specific log odds ratio of treatment k compared to baseline 1. For study arms receiving the baseline treatment (i.e. $k = b$) this simplifies to the study specific baseline term α_i.

The random effects model substitutes the constant treatment effect with the study specific treatment effect $\delta_{i,k}$. This is normally distributed with mean $mb_{i,k} = (\beta_{i,k} - \beta_{i,b})$ and variance σ^2, where σ^2 is the random effect variance and assumed to be constant across all treatment comparisons. The modifications for the random effects model are as follows:

$$\text{logit}(p_{i,k}) = \alpha_i + \delta_{i,k}$$

$$\delta_{i,k} \sim N(mb_{i,k}, \sigma^2)$$

$$mb_{i,k} = (\beta_{i,k} - \beta_{i,b})$$

Inconsistency Evaluation

Inconsistency evaluation was planned for any closed loops in the network of evidence that were informed by both direct and indirect evidence. However, there were no such loops so inconsistency evaluation was not necessary.

Supplementary table S9 – Full list of models

Target population	#	Model type	Population eligibility criteria*	Comparator eligibility criteria**
**Event-free survival**				
**Stage IB-IIIA**	**1**	**Base case**	**Base case**	**Base case**
Stage IB-IIIA	2	Sensitivity analysis 1 (population)	Expanded	Base case
Stage IB-IIIA	3	Sensitivity analysis 2 (comparator)	Base case	Expanded
**Stage IB-II**	**4**	**Base case**	**Base case**	**Base case**
Stage IB-II	5	Sensitivity analysis 1 (population)	Expanded	Base case
Stage IB-II	6	Sensitivity analysis 2 (comparator)	Base case	Expanded
**Stage IIIA**	**7**	**Base case**	**Base case**	**Base case**
Stage IIIA	8	Sensitivity analysis 1 (population)	Expanded	Base case
Stage IIIA	9	Sensitivity analysis 2 (comparator)	**Base case**	Expanded
**Stage IIIA N2**	**10**	**Base case**	**Base case**	**Base case**
Stage IIIA N2	11	Sensitivity analysis 1 (population)	Expanded	**Base case**
Stage IIIA N2	12	Sensitivity analysis 2 (comparator)	**Base case**	Expanded

**Stage IB-IIIA & PD-L1 ≥ 1%**	**13**	**PD-L1 specific**	**Base case**	**Base case**
**Stage II-IIIA & PD-L1 ≥ 1%**	**14**	**EMA target**	**Base case**	**Base case**
**Pathological complete response**				
**Stage IB-IIIA**	**15**	**Base case**	**Base case**	**Base case**
**Stage IB-II**	**16**	**Base case**	**Base case**	**Base case**
**Stage IIIA**	**17**	**Base case**	**Base case**	**Base case**
**Stage IIIA N2**	**18**	**Base case**	**Base case**	**Base case**
**Stage IB-IIIA & PD-L1 ≥ 1%**	**19**	**PD-L1 specific**	**Base case**	**Base case**
**Stage II-IIIA & PD-L1 ≥ 1%**	**20**	**EMA target**	**Base case**	**Base case**

**The base case NMA eligibility criteria for population was rNSCLC (i.e potentially resectable patients randomized prior to surgery or receipt of neoadjuvant therapy); this was expanded in a sensitivity analyses to include RCTs that randomized patients after surgical resection, and led to the inclusion of RCTs for which eligibility was restricted to patients who had a complete resection (R0).*
***The base case NMA eligibility criteria for the comparator was 3rd generation chemotherapies; this was expanded to include RCTs involving 2nd generation platinum-based chemotherapies.*
***Abbreviations**: EMA, European Medicines Agency.*

Supplementary material B: Results

Section B.1. Systematic review results

Supplementary figure S1 – PRISMA

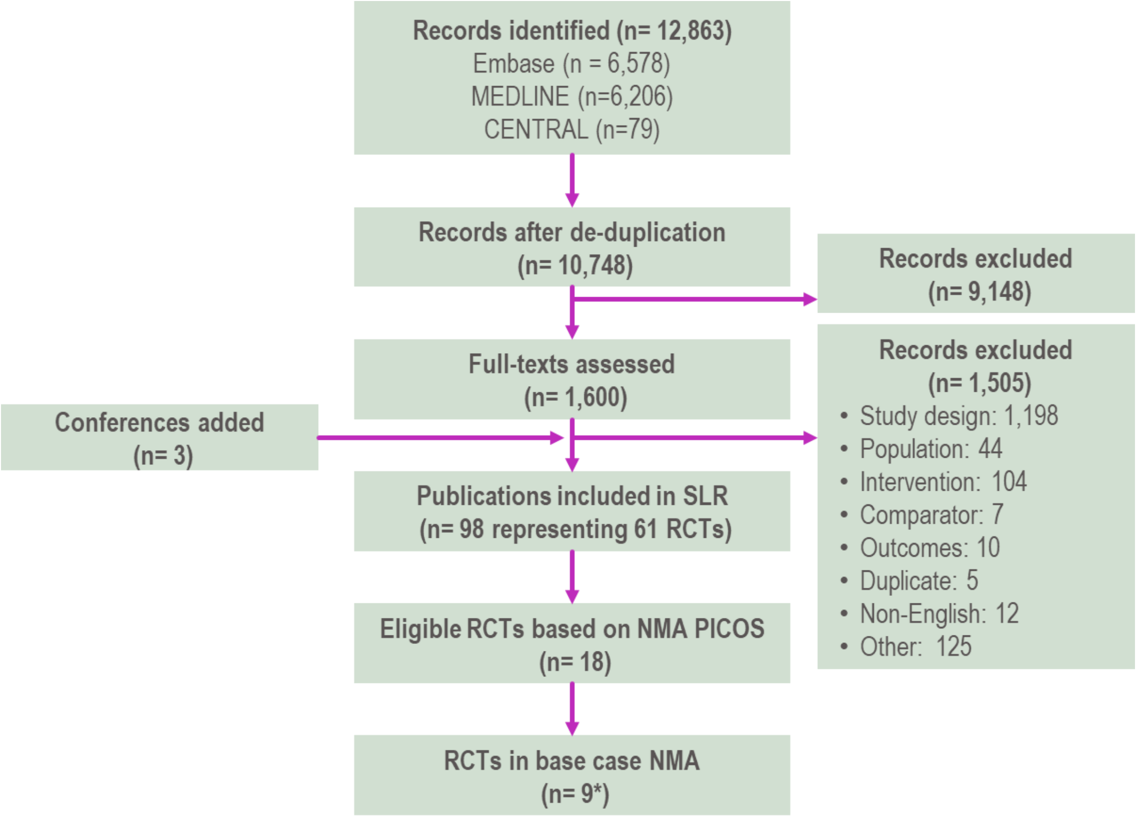

***The remaining nine studies were included in sensitivity analyses*

Supplementary figure S2 – Risk of bias assessment

Analysis	Study	Randomization process	Deviations from intended interventions	Missing outcome data	Measurement of the outcome	Selection of reported result	Overall
Base case studies	CHEST (Scagliotti 2012) [35]	Yellow	Yellow	Green	Green	Yellow	Yellow
	IFCT 0101 (Girard 2010) [38]	Yellow	Yellow	Green	Green	Yellow	Yellow
	SWOG S9900 (Pisters 2010) [36]	Yellow	Yellow	Green	Green	Yellow	Yellow
	NATCH (Felip 2010) [34]	Green	Yellow	Green	Green	Green	Yellow
	Li 2009 [37]	Yellow	Yellow	Green	Green	Yellow	Yellow
	SAKK 16/00 (Pless 2015) [40]	Green	Yellow	Green	Green	Yellow	Yellow
	WJTOG 9903 (Katakami 2012) [39]	Yellow	Yellow	Green	Green	Yellow	Yellow
	NADIM II (Provencio 2022) [33]	Green	Yellow	Green	Green	Yellow	Yellow
	CM816 (Forde 2022) [30, 32]	Green	Yellow	Green	Green	Green	Yellow
Sensitivity analyses (3rd and 2nd generation)	Chen 2013 [41]	Yellow	Yellow	Green	Green	Yellow	Yellow
	IFCT 0001 (Depierre 2002) [42]	Green	Yellow	Green	Green	Yellow	Yellow
	MRC LU22 (Gilligan 2007) [43]	Yellow	Yellow	Green	Green	Yellow	Yellow
	JCOG 9209 (Nagai 2003) [44]	Red	Yellow	Green	Green	Yellow	Red
	Rosell 1994 [45,46]	Green	Yellow	Green	Green	Yellow	Yellow
Sensitivity analyses (completely resected)	JBR10 (Winton 2005) [8,47]	Yellow	Yellow	Green	Green	Yellow	Yellow
	ANITA (Douillard 2006) [48]	Green	Yellow	Green	Green	Yellow	Yellow
	Ou 2010 [49]	Green	Yellow	Green	Green	Yellow	Yellow
	CALGB 9633 (Strauss 2008) [50]	Yellow	Yellow	Green	Green	Yellow	Yellow

***Legend**: Green: Low risk; Yellow: Some concerns; Red: High risk*

***Summary**:.Some studies poorly reported method of randomization and most trials were open-label, thus bias due to deviations from intended interventions was marked as yellow (some risk) for all trials. Note that Li 2009 had several concerns with their reporting in that their EFS KM curves did not start at 0.*

Section B.2. Proportional hazard assessments

Supplementary figure S3 – Kaplan-Meier curves of EFS for Base case: All stages, potentially resectable, 3rd generation chemotherapies

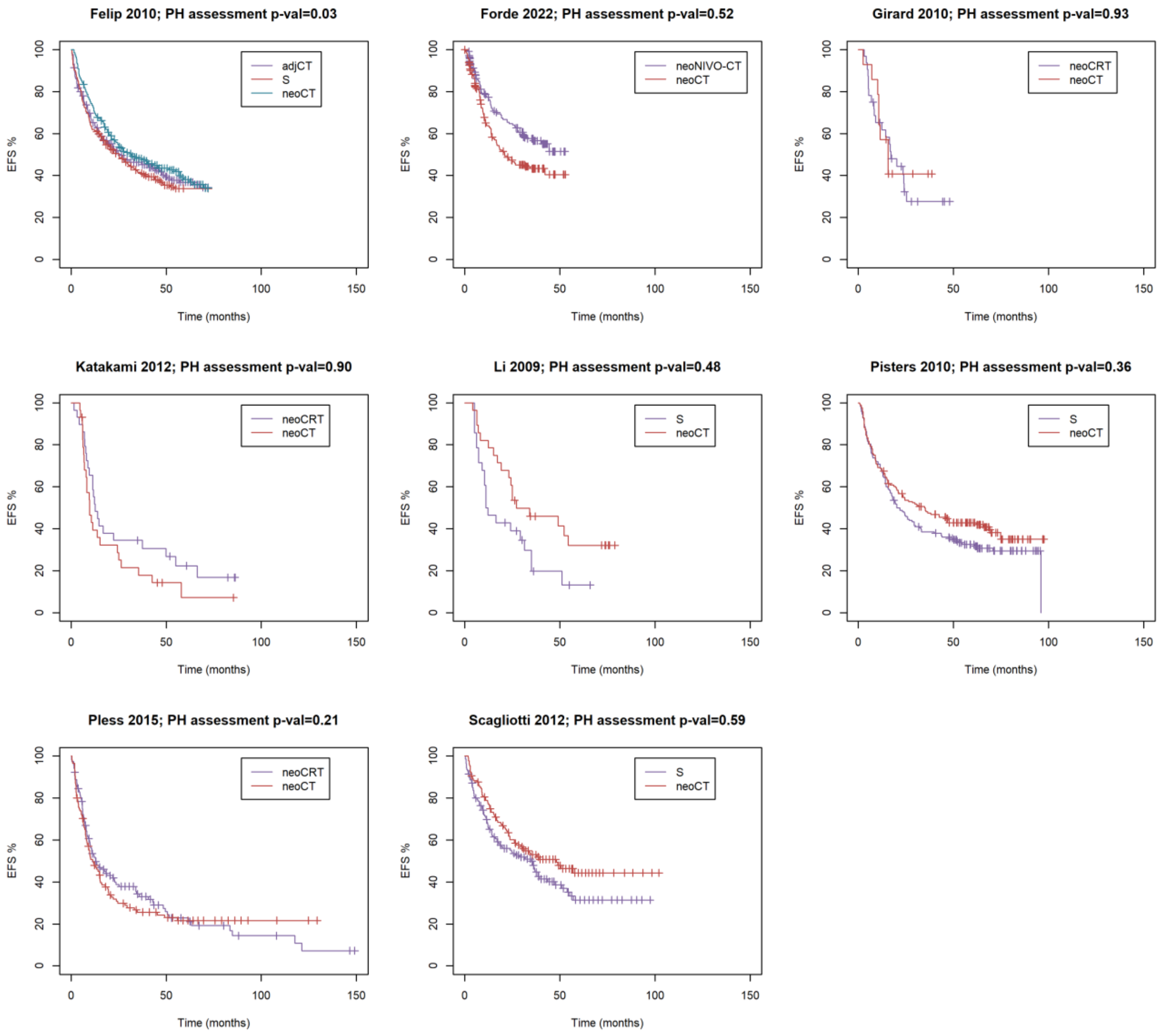

Explanation: *The only statistically significant violation of the PH assumption is found in Felip 2010 (p = 0.03), due to adjCT and S having a higher hazard than neoCT at the start, with the differences tapering off and the KM curves eventually meeting; however, the curves passed visual inspection as they had no obvious crossing or plateauing. While some of the KM curves cross, such as in Pless 2015 (SAK 16/00) and Girard 2010 (IFCT 0101), this is not necessarily due to a true PH violation and can be expected when curves have similar hazards and lie closely together. The confidence intervals for the HRs from Girard 2010 (IFCT 0101) and Pless 2015 (SAK 16/00) both span the equivalency value of one, thus indicating potentially equivalency in hazard between treatment curves in these studies. In Pisters 2010, the curves lie closely together to start, then begin to separate past one year. Though this suggests some potential deviation from PH, the difference was not detected as statistically significant (p=0.36).*

Note*: p-values reported are from the Grambsch-Therneau tests*

Abbreviations*: adjCT, adjuvant chemotherapy; neoCRT, neoadjuvant chemoradiotherapy; neoCT, neoadjuvant chemotherapy; neoNIVO+CT, neoadjuvant nivolumab-chemotherapy; S, surgery.*

Section B.3. Event-free survival

Supplementary table S10 – Data inputs and definitions for event-free survival in the base case

TRIAL (AUTHOR, YEAR)	OUTCOME DEFFINITION	ASSESSOR	COMPARISON	HR (95% CI)	HR SOURCE
**CM816 (Forde, 2022) [20,32]**	EFS: Time to recurrence, progression, or death; non-surgeries were counted as events if due to progression.	Blinded Independent Central Review	neoNIVO+CT vs. neoCT	0.68 (0.49, 0.93)	Author reported
**NATCH (Felip, 2010) [34]**	DFS: Time to recurrence, progression, or death; for those assessed as being unresectable at thoracotomy, time to intended surgery date.	NR	neoCT vs. S	0.92 (0.81, 1.04)	Author reported
			adjCT vs. S	0.96 (0.75, 1.22)	Author reported
			adjCT vs. neoCT	1.04 (0.86, 1.27)	Imputed*
**CHEST (Scagliotti 2012) [35]**	PFS: Time to recurrence, progression, or death	NR	neoCT vs. S	0.70 (0.50, 0.97)	Author reported‡
**SWOG S9900 (Pisters 2010) [36]**	PFS: Time to first progression or death	NR	neoCT vs. S	0.80 (0.61, 1.04)	Author reported
**Li 2009 [37]**	DFS: Time to recurrence, or time to surgery date for those with incomplete resection*	NR	neoCT vs. S	0.53 (0.28, 0.99)	Generated from pseudo-IPD**
**IFCT 0101 (Girard 2010) [38]**	PFS: Time to first progression or death	External multi-disciplinary monitoring committee	neoCRT vs. neoCT	1.10 (0.48, 2.50)	Generated from pseudo-IPD
**WJTOG 9903 (Katakami 2012) [39]**	PFS Time to progression***	NR	neoCRT vs. neoCT	0.68 (0.38, 1.21)	Author reported
**SAKK 16/00 (Pless 2015) [40]**	EFS: Time to relapse, progression, second tumour, or death	Local assessment	neoCRT vs. neoCT	0.91 (0.71, 1.25)	Author reported†

**Comparison between 2 intervention arms of 3-arm trial generated using method by Woods et al. 2010 [73].*
*** Non-standard presentation of KM curves in the publication (i.e., the curves did not begin at time 0).*
†Authors reported HR of 1.1, with unclear direction of effect. Inverse HR of 0.9 (= 1 / 1.1) for NeoCRT vs NeoCT inferred from digitization of KM curve. Authors were contacted for clarification but did not respond.
‡ Described by authors as being an adjusted HR (cofactor adjusted by baseline stage; no interaction term). Unadjusted HR not reported.
*********Although WJTOG 9903 and Li 2009 did not explicitly include death in their definitions, assessment of the KM curve indicates that death was indeed included as an event**.***
Abbreviations: *CT, chemotherapy; CRT, chemoradiotherapy; DFS, disease free survival; EFS, event free survival; HR, hazard ratio; neo, neoadjuvant; NIVO, nivolumab; NR, not reported; PFS, progression-free survival; S, surgery.*

Supplementary table S11 – Pairwise event-free survival hazard ratio estimates in the base case stage IB-IIIA model (fixed effect model estimates)

HR (95% CrI)	adjCT	neoCRT	neoNIVO+CT	S	neoCT
**adjCT**	--	1.17 (0.86, 1.60)	**1.52 (1.04, 2.20)**	0.89 (0.72, 1.12)	1.03 (0.85, 1.25)
**neoCRT**	0.85 (0.62, 1.16)	--	1.29 (0.86, 1.93)	**0.76 (0.58, 0.99)**	0.88 (0.69, 1.12)
**neoNIVO+CT**	**0.66 (0.45, 0.96)**	0.77 (0.52, 1.16)	--	**0.59 (0.42, 0.82)**	**0.68 (0.49, 0.94)**
**S**	1.12 (0.89, 1.42)	**1.32 (1.01, 1.72)**	**1.71 (1.22 , 2.39)**	--	**1.16 (1.04, 1.29**)
**neoCT**	0.97 (0.80, 1.18)	1.14 (0.89, 1.45)	**1.47 (1.07, 2.02)**	**0. 86 (0.78, 0.96)**	--

Note: *Estimates are comparing rows vs columns; bolded values indicate HRs are statistically significant.*
Abbreviations*: adj, adjuvant; CrI, credible interval, CT, chemotherapy; CRT, chemoradiotherapy; HR, hazard ratio; neo, neoadjuvant; NIVO, nivolumab; S, surgery.*

Supplementary table S12 – Event-free survival percentages at 2, 3, and 5 years anchored on neoCT in the base case models (fixed effect model estimates)

% (95% CrIs)	TIME (YEARS)	neoNIVO+CT	adjCT	neoCRT	S	neoCT
**STAGE IB-IIIA**	2	64 (52, 74)	51 (40, 60)	56 (45, 66)	46 (38, 55)	52 (44, 59)
	3	55 (42, 67)	41 (30, 51)	46 (34, 58)	36 (28, 46)	42 (34, 50)
	5	44 (30, 58)	29 (19, 40)	35 (23, 47)	25 (17, 34)	30 (22, 38)

STAGE IB-II	2	67 (44, 82)	64 (50, 76)	_	61 (47, 73)	65 (52, 76)
	3	58 (33, 77)	54 (39, 69)	_	51 (36, 66)	55 (41, 69)
	5	46 (20, 69)	42 (25, 60)	_	38 (23, 56)	43 (28, 60)
STAGE IIIA	2	63 (48, 75)	_	_	22 (5, 46)	45 (35, 54)
	3	54 (38, 68)	_	_	13 (2, 36)	34 (25, 44)
	5	43 (26, 59)	_	_	6 (0, 24)	23 (14, 32)
STAGE IIIA N2	2	63 (48, 75)	_	49 (36, 61)	_	45 (35, 54)
	3	54 (38, 68)	_	39 (26, 52)	_	34 (25, 44)
	5	43 (26, 59)	_	27 (15, 41)	_	23 (14, 32)

Note: *Survival estimates were generated using a lognormal survival curve fitted to the neoCT arm of CM816 as a reference.*
Abbreviations*: adj, adjuvant; CrI, credible interval, CT, chemotherapy; CRT, chemoradiotherapy; HR, hazard ratio; neo, neoadjuvant; NIVO, nivolumab; S, surgery.*

Supplementary figure S4 – Event-free survival hazard ratio estimates for neoNIVO+CT vs all relevant comparators across all models (base case and sensitivity models)

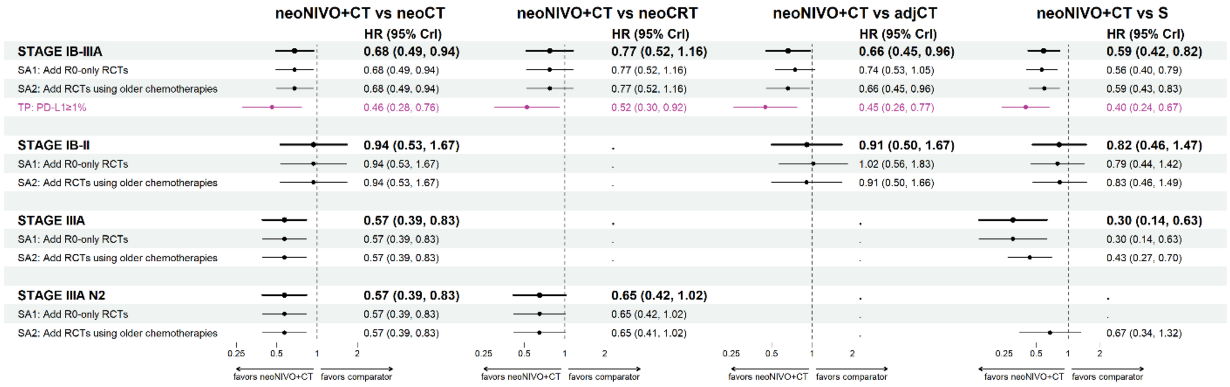

Abbreviations*: adj, adjuvant; CrI, credible interval; CT, chemotherapy; CRT, chemoradiotherapy; neo, neoadjuvant; NIVO, nivolumab; R0, completely resected; RCTs, randomized controlled trials; S, surgery; SA, sensitivity analyses; TP, target population.*

Supplementary figure S5 – Network of evidence for EFS models with studies included in the sensitivity analyses

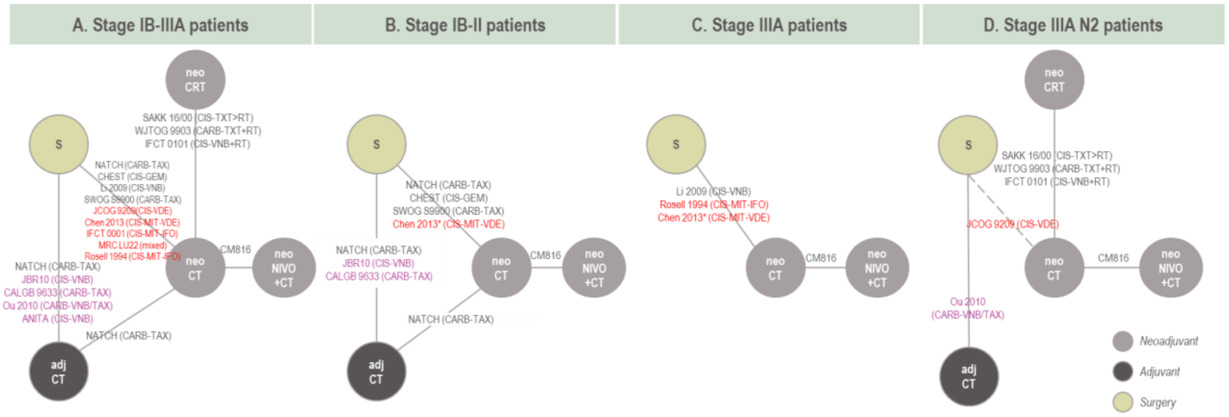

Note*: For studies involving neoCT, neoCRT, or S, see baseline characteristics regarding protocol-defined use of post-surgical therapy. Amongst the four base case studies which included a surgery alone arm, surgery was not followed by discretionary adjCT apart from Li 2009. Both Li 2009 and NATCH were also the only two studies that allowed for adjuvant radiotherapy following surgery in some cases. Studies with red font are those added in the sensitivity analyses on the comparator (expanding to 2nd generation chemotherapies), and studies in purple font are those added in the sensitivity analyses on the population (expanding to completely resected patients). Dotted line corresponds to a connection that only exists in sensitivity analysis..*
**Reported stage specific subgroups*
Abbreviations*: adj, adjuvant; CARB, carboplatin; CIS, cisplatin; CT, chemotherapy; CRT, chemoradiotherapy; GEM, gemcitabine; neo, neoadjuvant; NIVO, nivolumab; RT, radiotherapy; S, surgery; VNB, vinorelbine; TAX, paclitaxel; TXT, docetaxel*

Supplementary figure S6 – Event-free survival hazard ratio estimates for neoNIVO+CT vs all relevant comparators across the base case and the PD-L1 specific models (random effects model estimates)

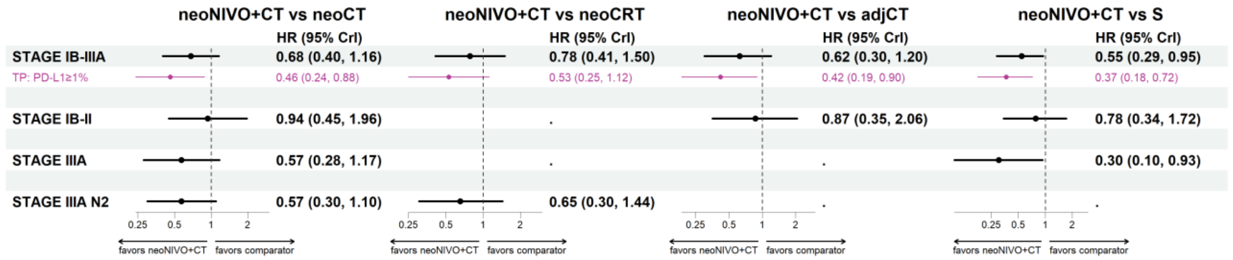

Note: *To estimate the between-study SD, a prior distribution uniform over 0.02 to 0.5 was used, which was based on the posterior distribution when using a vague prior in the largest available network across all base case and sensitivity analyses*
Abbreviations*: adj, adjuvant; CrI, credible interval, CT, chemotherapy; CRT, chemoradiotherapy; HR, hazard ratio; neo, neoadjuvant; NIVO, nivolumab; S, surgery.*

Section B.4. Pathological complete response

Supplementary table S13 – Data inputs and definitions for pathological complete response in the base case

TRIAL	OUTCOME DEFFINITION	TREATMENT	EVENTS	N	PERCENTAGE	DATA SOURCE
**CM816 (Forde 2022) [20,32]**	T0N0 (BIPR)	neoCT	4	179	2.2	Author reported
		neoNIVO+CT	43	179	24	Author reported
**IFCT 0101 (Girard 2010) [38]**	T0N0	neoCT	0	14	0	Author reported
		neoCRT	2	32	6.3	Author reported
**WJTOG 9903 (Katakami 2012) [39]**	T0N0	neoCT	0	29	0	Author reported
		neoCRT	3	29	10.3	Author reported
**SAKK 16/00 (Pless 2015) [40]**	Not reported	neoCT	11	115	9.6	Author reported
		neoCRT	16	117	13.7	Author reported
**NADIM II (Provencio 2022) [33]**	T0N0 (BIPR)	neoCT	2	29	6.9	Author reported
		neoNIVO+CT	21	57	36.8	Author reported

Abbreviations*: BIPR, blinded independent pathology review; CT, chemotherapy; CRT, chemoradiotherapy; neo, neoadjuvant; NIVO, nivolumab..*

Supplementary table S14 – Pathological complete response percentages anchored on neoCT in the base case models (fixed effect model estimates)

% (95% CrIs)	neoCRT	neoNIVO+CT	neoCT
**STAGE IB-IIIA**	3.5 (0.7, 10.4)	20.4 (4.3, 48.7)	2.2 (0.6, 4.5)
**STAGE IIIA**	--	13.9 (3.4, 52.0)	0.9 (0.4, 2.6)
**STAGE IIIA N2**	1.5 (0.4, 6.4)	18.6 (3.9, 67.0)	0.9 (0.4, 2.6)

Note: *Proportion estimates were generated using the neoCT arm of CM816 as a reference. The stage IB-II results are not represented in this table as they were only informed by CheckMate 816, as such, no NMA was conducted for this target population.*
Abbreviations*: adj, adjuvant; CrI, credible interval, CT chemotherapy; CRT, chemoradiotherapy; HR, hazard ratio; neo, neoadjuvant; NIVO, nivolumab.*

Supplementary table S15 – Pairwise pathological complete response odd ratio estimates in the base case stage IB-III model (fixed effect model estimates)

OR (95% CrI)	neoCRT	neoNIVO+CT	neoCT
**neoCRT**	--	**0.14 (0.04, 0.43)**	1.77 (0.84, 3.88)

**neoNIVO+CT**	**7.15 (2.31, 24.34)**	--	**12.53 (5.60, 33.82)**
**neoCT**	0.57 (0.26, 1.19)	**0.08 (0.03, 0.18)**	--

Note: *Estimates are comparing rows vs columns; bolded values indicate HRs are statistically significant.*
Abbreviations*: CrI, credible interval, CT, chemotherapy; CRT, chemoradiotherapy; OR, odds ratio; neo, neoadjuvant; NIVO, nivolumab; S, surgery.*

Supplementary figure S7 – Pathological complete response odd ratio estimates for neoNIVO+CT vs all relevant comparators across the base case and the PD-L1 specific models (random effects model estimates)

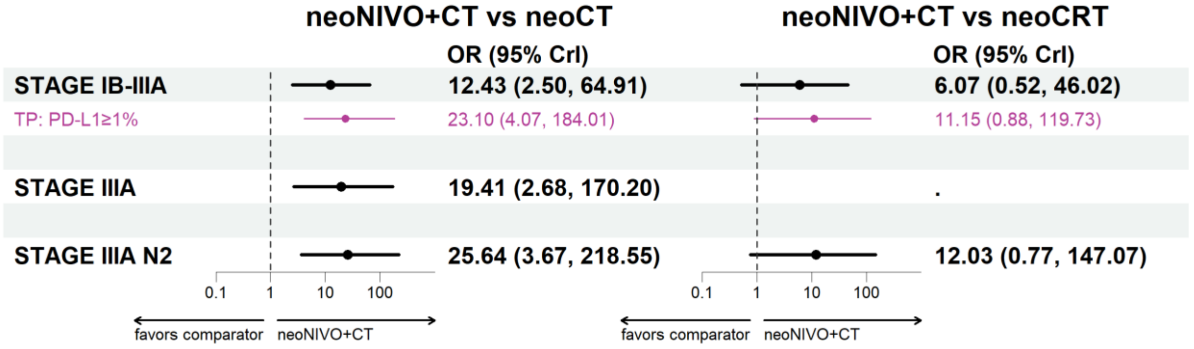

Note: *To estimate the between-study SD, a prior distribution uniform over 0 to 2 was used, which was based on the posterior distribution when using a vague prior in the largest available network across all base case and sensitivity analyses*
Abbreviations*: CrI, credible interval, CT, chemotherapy; CRT, chemoradiotherapy; neo, neoadjuvant; NIVO, nivolumab; OR, odds ratio; S, surgery.*

Section B.5. Stage II-IIIA, PD-L1 >=1% results

Supplementary table S16 – Baseline patient characteristics across EFS evidence base for trials included in the stage II-IIIA PD>=1% model

TRIAL	TREATMENT	SAMPLE SIZE	MEDIAN AGE (YEARS)	MALE (%)	ASIAN (%)	PERFORMANCE STATUS (%)		HISTOLOGY (%)	
						0	1	SQ	NSQ
**CM816**[20,32]**	neoCT	81	64	72	57	72	28	55	45
	neoNIVO+CT	86	64	77	56	73	27	52	48
**NATCH [34]**	S	210	64	88	NR	49*	50*	50	50†
	neoCT	199	65	88	NR	44*	54*	54	46†
**CHEST [35]**	adjCT	210	64	86	NR	45	53	49	51†
	neoCT	129	61	78	NR	74	26	37	63†
**Li 2009 [37]**	S	141	63	89	NR	70	30	45	55†
	neoCT	28	56	68	100	36	64	36	64†
**IFCT 0101 [38]**	neoCT	14		64	NR	71	29	57	43†
	neoCRT	17	56	88	NR	82	18	41	59†
	neoCRT	15		87	NR	73	27	60	40†
**WJTOG 9903 [39]**	neoCT	29	57	66	100	NR	NR	28	72†
	neoCRT	31	58	68	100	NR	NR	16	84†
**SAKK 16/00 [40]**	neoCRT	117	60	67	NR	71	29	36	64†
	neoCT	115	59	67	NR	69	31	31	69†

***Data taken from PD-L1 ≥1% and stage II-IIIA.*
**NATCH was the only study that included patients with a performance status of 2 (n <1%)*
† Inferred from proportion with squamous cell histology
Abbreviations*: CT, chemotherapy; CRT, chemoradiotherapy; neo, neoadjuvant; NIVO, nivolumab; NR, not reported; NSC, non squamous cell; S, surgery; SC, squamous cell.*

Note: *SWOG S9900 was conducted amongst patients with stage IB – II (T3N1) disease, yet did not include subgroup data for the stage II population; as such, it was not included in this model.*

Supplementary table S17 – EFS NMA inputs evidence base for stage II-IIIA PD>=1% model

TRIAL	STAGE	SUBGROUP	COMPARISON	OUTCOME	EFS HR (95% CI)	HR SOURCE
**CM816 [20,32]**	II-IIIA	PD-L1≥1%	neoNIVO+CT vs. neoCT	EFS	0.49 (0.29, 0.83)	Data on file (subgroup data)
**NATCH [34]**	II & T3N1	--	neoCT vs. S	DFS	0.81 (0.64, 1.02)	Author reported (subgroup data)
			adjCT vs. S	DFS	0.87 (0.54, 1.38)	Author reported (subgroup data)
			adjCT vs. neoCT	DFS	1.07 (0.73, 1.57)	Imputed (subgroup data)*
**CHEST [35]**	IIB & T3N1	--	neoCT vs. S	PFS	0.51 (0.32, 0.80)	Author reported (subgroup data)
**Li 2009 [37]**	IIIA	--	neoCT vs. S	DFS	0.53 (0.28, 0.99)	Generated from pseudo-IPD**
**IFCT 0101 [38]**	IIIA N2	--	neoCRT vs. neoCT	PFS	1.10 (0.48, 2.50)	Generated from pseudo-IPD
**WJTOG 9903 [39]**	IIIA N2	--	neoCRT vs. neoCT	PFS	0.68 (0.38, 1.21)	Author reported
**SAKK 16/00 [40]**	IIIA N2	--	neoCRT vs. neoCT	EFS	0.91 (0.71, 1.25)	Author reported†

Abbreviations: *CT, chemotherapy; CRT, chemoradiotherapy; DFS, disease free survival; EFS, event free survival; HR, hazard ratio; neo, neoadjuvant; NIVO, nivolumab; NR, not reported; PFS, progression-free survival; S, surgery.*
**Comparison between 2 intervention arms of 3-arm trial generated using method by Woods et al. 2010.*
*** Non-standard presentation of KM curves in the publication (i.e., the curves did not begin at time 0)*
†Authors reported HR of 1.1, with unclear direction of effect. Inverse HR of 0.9 (= 1 / 1.1) for NeoCRT vs NeoCT inferred from digitization of KM curve. Authors were contacted for clarification but did not respond.

Supplementary figure S8 – Event-free survival hazard ratio estimates for neoNIVO+CT vs all relevant comparators for stage II-IIIA PD>=1% model (fixed effect model estimates)

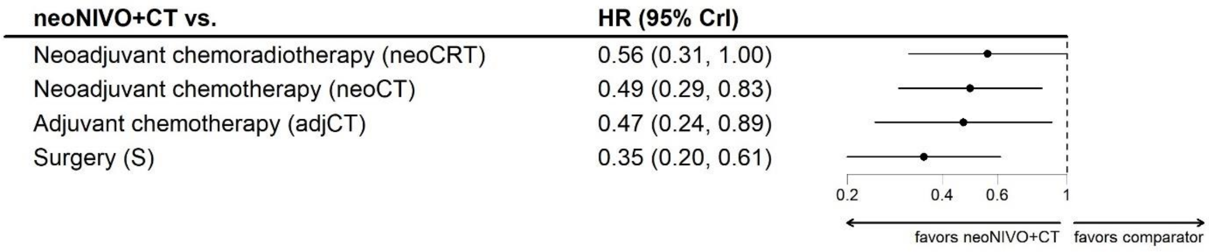

Supplementary figure S9 – Event-free survival hazard ratio estimates for neoNIVO+CT vs all relevant comparators for stage II-IIIA PD>=1% model (random effects model estimates)

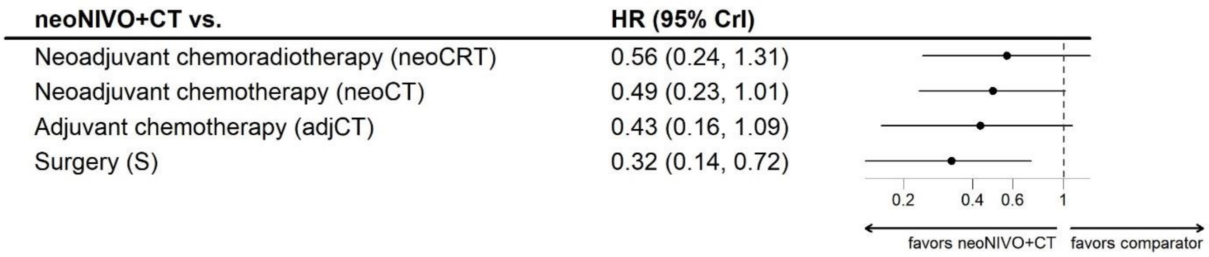

Note: *The random effects model was informed as a uniform distribution ranging from 0.02 to 0.5 (U[0.02, 0.5]).*

Supplementary table S18 – Pathological complete response NMA inputs evidence base for stage II-IIIA PD>=1% model

TRIAL	STAGE	SUBGROUP	OUTCOME DEFFINITION	TREATMENT	EVENTS	N	DATA SOURCE
**CM816 [20,32]**	II-IIIA	PD-L1≥1%	T0N0 (BIPR)	neoCT<br>neoNIVO+CT	2<br>26	86<br>81	IA2 patient-level data (subgroup data)<br>IA2 patient-level data (subgroup data)
**IFCT 0101 [38]**	IIIA N2		T0N0	neoCT<br>neoCRT	0<br>2	14<br>32	Author reported<br>Author reported
**WJTOG 9903 [39]**	IIIA N2		T0N0	neoCT<br>neoCRT	0<br>3	29<br>29	Author reported<br>Author reported
**SAKK 16/00 [40]**	IIIA N2		Not reported	neoCT<br>neoCRT	11<br>16	115<br>117	Author reported<br>Author reported
**NADIM II [33]**	IIIA	PD-L1≥1%	T0N0 (BIPR)	neoCT<br>neoNIVO+CT	1<br>16	15<br>30	Patient-level data (subgroup data)<br>Patient-level data (subgroup data)

***Abbreviations**: BIPR, blinded independent central review; CT, chemotherapy; CRT, chemoradiotherapy; neo, neoadjuvant; NIVO, nivolumab..*

Supplementary figure S10 – Pathological complete response odds ratio estimates for neoNIVO+CT vs all relevant comparators for stage II-IIIA PD>=1% model (fixed effect model estimates)

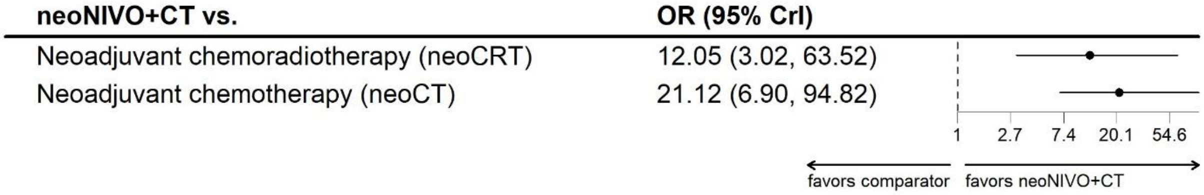

***Abbreviations**: CT, chemotherapy; OR, odds ratio; neo, neoadjuvant; NIVO, nivolumab..*

Note, random effect pCR model estimates for neoNIVO+CT vs all relevant comparators in the stage II-IIIA PD>=1% model were not run as the data was too sparse so the model did not converge.

<u>Section B.6. Tabular summary of adverse events</u>

Supplementary table S19 – Proportion of patients with grade 3 to 4 adverse events

TRIAL	DEFINITION	INTERVENTION DESCRIPTION	COMPARATOR DESCRIPTION	INTERVENTION n/N (%)	COMPARATOR n/N (%)
**CM816 [20,32]**	Grade 3-4 treatment-related AEs	neoNIVO+CT	neoCT	63/176 (35.8)	67/176 (38.1)
**CHEST [35]**	Any grade 3-4 AEs	neoCT	S	52/127 (41)	15/136 (11)
**SAKK 16/00 [40]**	Grade 3-4 related to CT	neoCRT	neoCT	49/110 (45)	73/121 (60)
	Grade 3-4 AEs related to RT	neoCRT	neoCT	9/98 (9)	Not applicable

***Abbreviations**: AE, adverse events; CRT, chemoradiotherapy; CT, chemotherapy; n, number of patients in the sample; N, number of patients in the population; NIVO, nivolumab; neo, neoadjuvant; RT, radiotherapy; S, surgery.*

Supplementary table S20 – Proportion of patients with AEs leading to treatment discontinuation

TRIAL	DEFINITION	INTERVENTION DESCRIPTION	COMPARATOR DESCRIPTION	INTERVENTION n/N (%)	COMPARATOR n/N (%)
**CM816 [20,32]**	Any grade AE leading to discontinuation	neoNIVO+CT	neoCT	18/176 (10)	20/176 (11)
	Grade 3-4 AE leading to discontinuation	neoNIVO+CT	neoCT	10/176 (6)	7/176 (4)

	Any grade treatment-related AE leading to discontinuation	neoNIVO+CT	neoCT	18/176 (10)	17/176 (10)
	Grade 3-4 treatment-related AE leading to discontinuation	neoNIVO+CT	neoCT	10/176 (6)	6/176 (3)
**SAKK 16/00 [40]**	Treatment related AE leading to permanent discontinuation of CT	neoCRT	neoCT	5/117 (4)	7/115 (6)

Abbreviations: AE, adverse event; CRT, chemoradiotherapy; CT, chemotherapy; n, number of patients in the sample; N, number of patients in the population; NIVO, nivolumab; neo, neoadjuvant; S, surgery

Woods, B.S., Hawkins, N. & Scott, D.A. Network meta-analysis on the log-hazard scale, combining count and hazard ratio statistics accounting for multi-arm trials: A tutorial. *BMC Med. Res. Methodol*. 2010, 10, 54. https://doi.org/10.1186/1471-2288-10-54
